# Current aspects of small extracellular vesicles in pain process and relief

**DOI:** 10.1186/s40824-023-00417-3

**Published:** 2023-08-10

**Authors:** Lanyu Zhang, Jin Liu, Cheng Zhou

**Affiliations:** 1 Department of Anesthesiology, West China Hospital, Sichuan University, Chengdu, China; 2 Laboratory of Anesthesia & Critical Care Medicine, National-Local Joint Engineering Research Centre of Translational Medicine of Anesthesiology, West China Hospital of Sichuan University, Chengdu, 610041 Sichuan China

**Keywords:** Extracellular vesicle, Pain, Neuron, Mesenchymal stem cell

## Abstract

Small extracellular vesicles (sEVs) have been identified as a noteworthy paracrine mechanism of intercellular communication in diagnosing and managing neurological disorders. Current research suggests that sEVs play a pivotal role in the pathological progression of pain, emphasizing their critical function in the pathological progression of pain in acute and chronic pain models. By facilitating the transfer of diverse molecules, such as proteins, nucleic acids, and metabolites, sEVs can modulate pain signaling transmission in both the central and peripheral nervous systems. Furthermore, the unique molecules conveyed by sEVs in pain disorders indicate their potential as diagnostic biomarkers. The application of sEVs derived from mesenchymal stem cells (MSCs) in regenerative pain medicine has emerged as a promising strategy for pain management. Moreover, modified sEVs have garnered considerable attention in the investigation of pathological processes and therapeutic interventions. This review presents a comprehensive overview of the current knowledge regarding the involvement of sEVs in pain pathogenesis and treatment. Nevertheless, additional research is imperative to facilitate their clinical implementation.

**Graphical Abstract:**

Schematic diagram of sEVs in the biogenesis, signal transmission, diagnosis, and treatment of pain disorders. Small extracellular vesicles (sEVs) are secreted by multiple cells, loading with various biomolecules, such as miRNAs, transmembrane proteins, and amino acids. They selectively target other cells and regulating pain signal transmission. The composition of sEVs can serve as valuable biomarkers for pain diagnosis. In particular, mesenchymal stem cell-derived sEVs have shown promise as regenerative medicine for managing multiple pain disorders. Furthermore, by modifying the structure or contents of sEVs, they could potentially be used as a potent analgesic method.

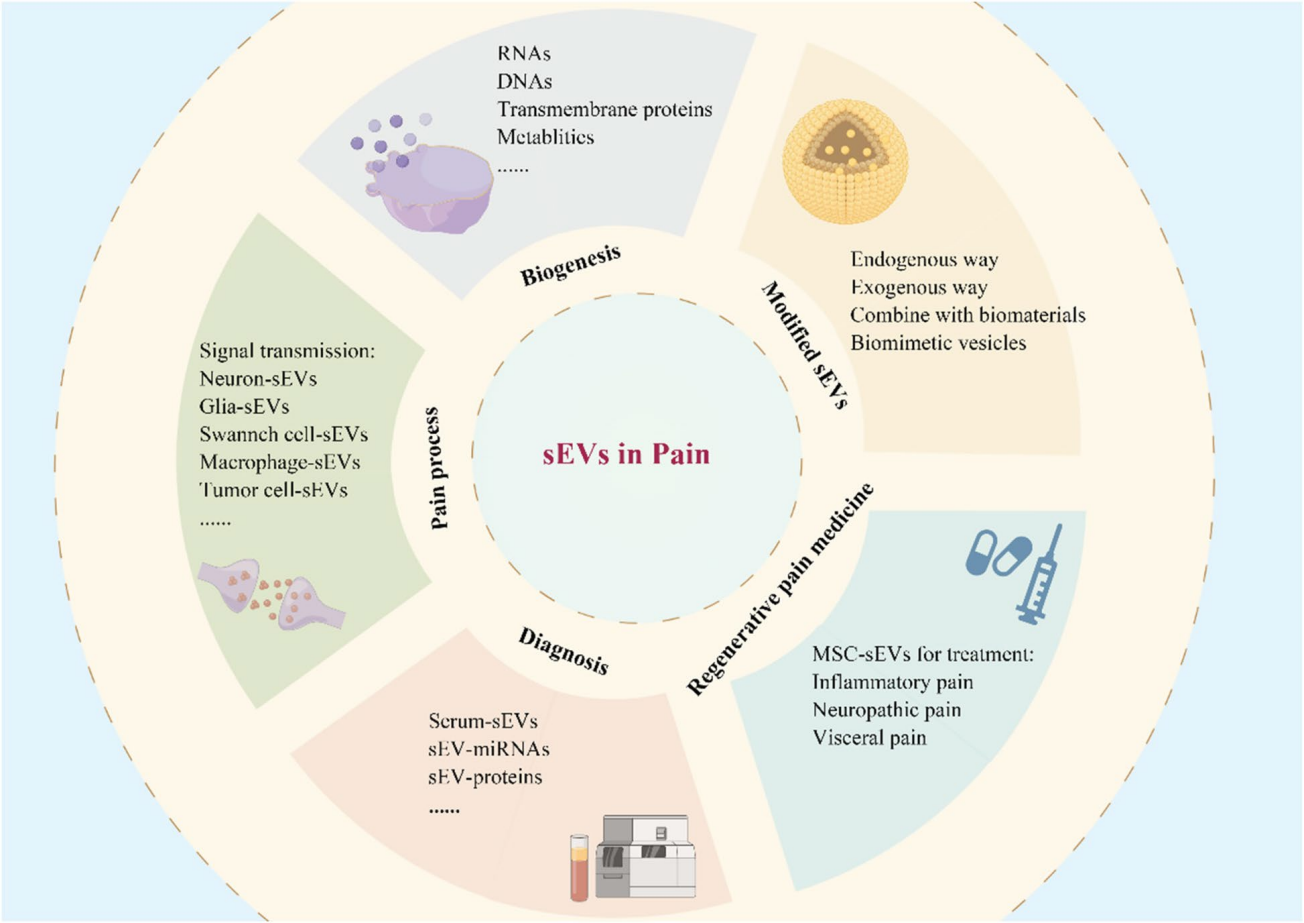

## Introduction

The International Association for the Study of Pain (IASP) defines pain as an unpleasant sensory and emotional experience associated with, or resembling that associated with, actual or potential tissue damage [1]. The sensation of acute pain serves as an imperative biological warning mechanism that notifies individuals of potential threats or damage. However, if the underlying cause of the pain signal remains unresolved, it may progress into chronic pain over time. Chronic pain is a significant contributor to human affliction and disability, necessitating recurrent medical attention. Current investigations categorize chronic pain as nociceptive, neuropathic, and nociplastic, based on a novel classification system. This revised approach is founded on the concept that pain mechanisms can be stratified according to their underlying pathophysiology, allowing for more targeted and effective therapeutic interventions [2]. The nociceptive pain is from tissue injury, including somatic and visceral damage, such as osteoarthritis, ischemia, inflammatory bowel disease (IBD), and tumor infiltration et al. The second category refers to neuropathic pain, which primarily occurs due to nerve damage in either the central or peripheral nervous system. Examples of such conditions that can cause neuropathic pain mainly include neurodegenerative diseases, spinal cord injury (SCI), and nerve compression, among others [3]. The nociplastic pain is caused by a sensitized nervous system, including diffuse sensitization, and functional visceral pain et al. [2, 4]. Even though pain has multiple etiologies and taxonomies, nociceptive signaling pathways are implicated in most of them. It is crucial to understand the mechanism behind pain signal transduction to understand the origins of pain; and how it progresses from acute to chronic. Recent research has identified small extracellular vesicles (sEVs) as essential mediators of paracrine signaling between cells during pain transduction. The presence of extracellular vesicles (sEVs) released by cells is widely observed in diverse intercellular communication processes. These sEVs originate from various sources, including the nervous system (neurons and glial cells), peripheral tissue cells, body fluids, and immune cells. Moreover, these vesicles display discernible attributes and exert various impacts in diverse physiological and pathological contexts. These vesicles possess distinctive characteristics and exert diverse effects in both physiological and pathological contexts. The production and characteristics of sEVs are also influenced by varying conditions [5].

sEVs are primarily sourced from cellular secretion and function as ubiquitous forms of paracrine communication among cells. They have emerged as a promising mode of biomolecule transportation for the modulation of gene expression or function, both over short and long distances. The transfer of cargo molecules between injured and recipient cells via sEVs holds immense potential in reprogramming the phenotype of the latter, thereby enabling them to acquire new functions. The release of sEV-associated signaling molecules from different cellular sources enables long-range transportation to remote target tissues via systemic circulation or lymphatic drainage. Thus, this network of sEV-mediated molecular signaling plays an indispensable role in maintaining homeostasis and the proper functioning of multiple organ systems. Selective encapsulation of specific cargo molecules within EVs facilitates precise regulation of inter-tissue interactions, thus achieving fine-tuned control over physiological and pathological processes. Moreover, the protective nature of sEVs shields their cargo molecules from degradation and dilution, facilitating efficient transmission of molecular information between cells and tissues.

The distinctive attributes of sEVs have rendered them a subject of immense exploration across diverse fields of physiological and pathological processes, such as cancer, neurodegenerative diseases, and cardiovascular disorders [6]. More recently, there has been a notable increase in attention paid to sEVs in the pain process. In line with this, the objective of this review is to deliver an exhaustive coverage of the progressions made in deciphering the role of sEVs in pain mechanisms, coupled with their prospective utility in clinical applications for diagnostic and therapeutic purposes.

### The biogenesis, characteristics, and isolation of sEVs

#### The biogenesis of sEVs

SEVs encompass a variety of subtypes, defined by their biogenesis and size. They have previously been categorized into three separate types, namely exosomes (Exos), microvesicles (MVs), and apoptotic bodies (ABs) [7]. Among them, Exos range in size from 30 to 150 nm, while MVs span from 50 to 1000 nm [7]. In comparison, apoptotic bodies, which arise from programmed cell death, exhibit significantly larger diameters exceeding 1000 nm [6]. The formation of these sEVs is mediated by distinct mechanisms involving intracellular membrane trafficking pathways. The biogenesis of exosomes is accomplished by the invagination of the plasma membrane or Golgi apparatus, resulting in the formation of intraluminal vesicles (ILVs) within multivesicular bodies (MVBs) in a sequential manner. It is worth mentioning that the membrane of EVs is derived from either plasma or endosome membranes, with plasma-derived EVs being reported to exhibit a 5-fold increase in efficiency compared to those originating from the endosome membranes [8]. These MVBs subsequently merge with the cellular membrane, ultimately culminating in the release of exosomes. This process requires several steps facilitated by endosomal sorting complexes (ESCRT)-dependent and -independent pathways [9, 10]. The ESCRT machinery, composed of four protein complexes (ESCRT-0, -I, -II, and -III) and an accessory Vps4 complex, plays a crucial role in this process. Conversely, MVs are generated through outward pinching of the plasma membrane, enclosing nearby biomolecules. The current subtyping methodology for EVs falls short of capturing the full spectrum of their characteristics. In recognition of this inadequacy, the MISEV2018 guideline offers a refined definition for EV subtype classification, which takes into account not only size but also biochemical composition, surpassing the former three-type classification system [11]. Despite this, there persists a marked bias towards exosomes in research studies, prompting us in this review to employ the term “small extracellular vesicles” as a more inclusive descriptor of this heterogeneous group. The presence of small extracellular vesicles (sEVs) ranging from 30 to 200 nm is prevalent, primarily consisting of exosomes and MVs. These subsets of vesicles are currently the most researched population [11].

**Fig. 1:**
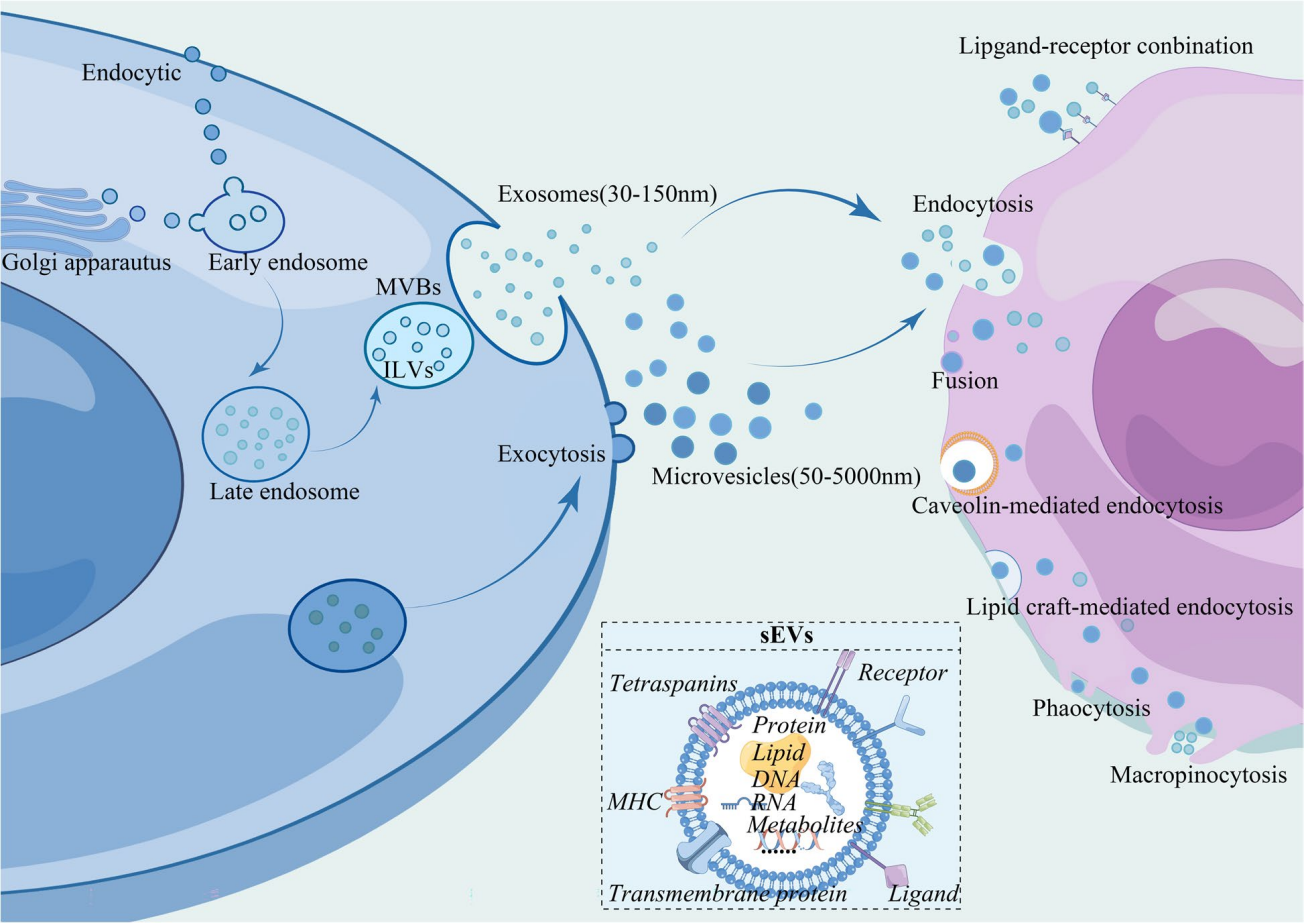
sEVs biogenesis and uptake. sEVs are formed by creating early endosomes, which then progress to late endosomes. Various contents, including nucleic acids and proteins, are loaded into the sEVs during this process. MVBs fuse with the cell membrane upon completion to release the small EVs, while larger EVs can be directly exocytosed. These sEVs target other cells through diverse pathways, primarily via fusion or endocytosis. MVBs: multivesicular bodies; sEV: small extracellular vesicle

 SEVs are characterized by their rich and diverse composition (Fig. 1). All types of sEVs have been shown to encompass a wide range of components including proteins, nucleic acids (such as DNA, microRNA, lncRNA), lipids, metabolites, as well as amino acids derived from the parent cells. The identification of distinct subtypes of sEVs lacks specificity due to the absence of precise markers. In this regard, non-tissue-specific membrane markers such as tetraspanins (i.e., CD63, CD81, CD9), MHC molecules, integrin, ligands, receptors, and flotillins are commonly used. Meanwhile, lumen proteins including TSG101, ALIX, HSPs, syntenin, and RAB GTPases, and lipids like ceramide, phosphatidylserine, sphingomyelin, and cholesterol have also been identified [12, 13]. Ceramide, phosphatidylserine, sphingomyelin, cholesterol, and others are among the lipids found in sEVs [12]. The generation of ceramide lipids through the hydrolysis of neutral type II sphingomyelins is essential for the transport of multivesicular bodies (MVBs) and the biogenesis of sEVs [14]. Additionally, phosphoinositides, which are membrane phospholipids involved in regulating membrane dynamics, contribute to sEVs release [15]. Despite the wide expression of molecules, there is still a lack of specific markers that can uniquely identify different subtypes of sEVs.

#### The uptake of sEVs

SEVs, upon release from their parent cells, traverse short or long distances to deliver their cargoes to target cells. These cargoes are instrumental in inducing phenotypic changes in the recipient cells, thereby influencing physiological or pathological processes. The internalization of sEVs by target cells involves a wide range of mechanisms. In particular, the proteins and glycoproteins presented on the surfaces of both sEVs and target cells are critical in determining the uptake mechanism. The proteins responsible for the binding process can be broadly categorized into several groups, namely tetraspanins, lectins, integrins, and scaffold proteins. Tetraspanins contributed to the selective target binding [16]. Integrins serve as transmembrane proteins that function as receptors for extracellular matrix proteins, including laminin and fibronectin [17]. Lectins located along the plasma membranes of cells, as well as proteoglycans present on the surface of sEVs, play a role in facilitating the docking process of sEVs [17, 18]. On the other hand, there are some special molecules that sEVs carried from parent cells, which are capable to combine to ligands on the specific targeted cells. The membranes of sEVs contain a diverse array of proteins, including a substantial number of G protein-coupled receptors (GPCRs). The receptors play a crucial role in acting as anchors for sEVs and facilitating their internalization through receptor-mediated uptake [19]. For example, leukocyte-derived sEVs expressed B1-kinin receptors in patients with vasculitis. These sEVs band to functional target cells via the B1-kinin receptor [20]. Moreover, the CD200R present on macrophage-derived sEVs interacts with the iSec1 ligand found on neurons, facilitating the transmission of mitochondria [21]. There has been more research conducted in the field of tumors. For example, the caveolin-1 on the sEVs increased and led to lung metastasis by regulating the expression of pre-metastatic niche marker genes and inflammatory chemokines in lung epithelial cells [22]. It was reported that the membrane proteins of sEVs had changed in tumor tissues, and the changed membrane proteins were able to be biomarkers for the diagnosis of the disorder [23, 24]. Moreover, based on the selection of these membrane proteins on sEVs, they were engineered to get a therapeutic effect as we have discussed in the part of *Engineered sEVs*. Numerous studies have been conducted regarding the engineering modification of membrane proteins on sEVs to enhance the targeting capability for disease treatment [25]. For a more comprehensive understanding of the ligand-receptor interaction, the published reviews on sEVs transportation and uptake deserve these references [17, 18].

Notably, fusion and endocytosis are the common modes of interaction between the EVs and the targeted cells [26]. Membrane fusion is believed to be facilitated by soluble N-ethylmaleimide sensitive factor attachment protein receptor (SNARE) complex. The formation of the four corresponding SNAREs establishes a robust linkage between the two lipid membranes for fusion [27]. For example, the SNARE on the membrane of neurons regulated the uptake of mesenchymal stem cells (MSCs)-derived sEVs [28]. Endocytosis, which encompasses various pathways including caveolin-mediated uptake, clathrin-dependent method, macropinocytosis, phagocytosis, and lipid raft-mediated internalization, is also significant [29]. The uptake mechanism of sEVs can vary depending on the specific cell type being targeted and the origin of these vesicles. For instance, it was observed that neurons employ selective clathrin- and dynamin-dependent endocytosis to internalize the sEVs derived from oligodendrocytes [30]. In contrast, the transfer of sEVs by oligodendrocytes to microglia selectively occurred via micropinocytosis [31] (Fig. 1).

After internalization by target cells, sEVs can elicit alterations in downstream signaling pathways through two distinct mechanisms: (1) direct contact *via* their surface receptors/ligands to trigger signaling pathway in the cells as mentioned above; (2) the delivery of bioactive molecules into target cells facilitates the alteration of expressions of signaling molecules [19]. The ligand-receptor mechanism and vesicle-loading method have already been employed to investigate the impact of sEVs on pain, as discussed in the following sections.

#### The isolation of sEVs

Currently, there is no single gold standard method to isolate sEVs. There are numerous methods to isolate sEVs, encompassing differential ultracentrifugation (DUC), density gradient centrifugation (DC), ultrafiltration, size-exclusion chromatography (SEC), combined multiple methods, commercial isolation kits et al. [7, 32–34]. Recent years have uncovered novel or modified approaches for isolation and purification, like the magnetic bead-mediated selective adsorption strategy and microfluidics [35]. Nevertheless, differential ultracentrifugation, density gradient centrifugation, and SEC continue to be the most frequently employed techniques for sEVs isolation and purification. It is worth noting that nearly all the above-mentioned separation techniques have limitations in achieving high yield and purity of sEVs concurrently (Table 1). The DUC method is the most commonly used technique for isolating sEVs. This method relies on employing different gradients during initial centrifugation steps, ultimately leading to ultra-high-speed centrifugation at speeds exceeding 100,000 g. The ultra-high-speed centrifugation technique necessitates the use of a corresponding ultracentrifuge and centrifuge tubes. The process of gradient centrifugation resulted in the disruption of a portion of the vesicles. This method achieved a moderate purity and production of sEVs from extracted samples [36, 37]. The centrifugation process of density gradient centrifugation involving a density gradient utilizes separation media, such as sucrose and iodixanol, in conjunction with DUC. This method enables the acquisition of sEVs that demonstrate improved purity, allowing them to segregate within specific gradient layers presented in the solution. But the yield is lower than DUC [37]. SEC employed the distinct velocities at which particles of varying sizes traverse porous polymer gel fillers, thereby facilitating the separation and isolation of vesicles according to their respective sizes [34]. The ultrafiltration method can obtain much larger vesicles by sequential filtration, but the production is low purity [38]. Immunomagnetic separation involves using antibodies and magnetic beads to capture sEVs. This method helps recognize and bind specific target antigens on the surface of sEVs to isolate vesicles [39]. Getting highly pure sEVs using this method is effective but expensive. However, it’s important to note that there is a widely accepted specific antigen on the surface of sEVs. Microfluidic technology uses small channels and fluid dynamics principles to control the flow of fluids. It allows for the separation and capture of sEVs by manipulating pressure, velocity, and direction within these channels [40]. However, the utilization of this methodology requires the use of specialized equipment to successfully acquire and maintain stable vesicles. There also exists alternative commercial kits for the isolation, although comprehensive elucidation of mechanisms remains unknown. These kits are characterized by their simple operational procedure. Nonetheless, the quality of sEVs obtained using these kits is different significantly [41]. The basic methods for characterization detection are including morphology by electron microscopy, size distribution by nanoparticle tracking analysis, protein marker analysis by western blotting, immunofluorescence, or flow cytometry. Moreover, due to the inherent heterogeneity of sEVs, the isolation methodology can significantly impact their components and function. There is an urgent requirement for a comprehensive and systematic approach to assessing the yield and purity of methods employed for acquiring sEVs, while also establishing a universally accepted extraction method as the gold standard.

**Table 1:** Isolation methods of sEVs

Methods		Process	Advantages	Disadvantages	Ref.
Centrifugation	Differential ultracentrifugation	400×g, 2000×g, 10,000×g centrifugation, and then ultracentrifugation of more than 100,000×g to obtain sEVs	Simple process, moderate production	Required expensive device, impurity, time-consuming	[36, 37]
	Density gradient centrifugation	DUC combines with separation media	High purity	Complicated process, low yield	[37]
Vesicle-size separation	Size-exclusion chromatography	Vesicles of different sizes were separated by different velocities in the filling process of porous polymer gels	High purity	Suitable for small-volume liquid, low yield	[34]
	Ultrafiltration	Appropriate pore size is used to eliminate larger cell debris and cells, thereby obtaining sEVs on the membrane	High yield, simple process, rapid	Low purity, larger vesicles	[38]
Immunoaffinity isolation	Immunomagnetic separation	The antibodies on beads combine with antigens on vesicles to capture sEVs	Specific vesicles, high purity	Expensive, low yield	[39]
Device	Microfluidic	precise control of samples and vesicle collection through miniaturized devices and microvalves, facilitating dynamic manipulation and isolation of sEVs	Precise control, stable yield, automation process	Expensive microfluidic device, low-volume sample	[40]
Others	Commercial kits	According to the commercial protocol	Simple process	Expensive, unstable quality	[41]

*sEVs *small extracellular vesicles, *DUC *Differential ultracentrifugation

### The sEVs in the nervous system for the pain process

Assorted conclusive evidence has illustrated the crucial function of sEVs in facilitating intercellular communication among varying neural cell categories in both physiological and pathological states. An advantage of sEVs is their ability to cross the blood-brain barrier (BBB) and blood-spinal cord barrier (BSB), thereby enabling access to remote targets throughout the nervous system. The recent progress in comprehension has illuminated the interplay among neurons, glial cells, immune cells, and tissue cells, all of which are engaged in managing pain signaling and its sustenance [42, 43]. Diverse kinds of sEVs derived from both the central and peripheral tissues have been associated with these mechanisms (Table 2) [44].

**Table 2:** sEVs in pain mechanisms

Published year	Condition	sEV type	Mechanisms	Effects	Ref.
2022	Carrageenan-induced pain	CD200R^+^ macrophage-derived sEVs	CD200R^+^ sEVs transferred mitochondria to neurons in DRG	Resolved transient inflammatory pain	[21]
2021	CIBP	Cancer cell-derivd sEVs	Increased let-7d-5p in sEVs inhibited OPRM1 in DRG	Induced bone pain	[111]
2017, 2021	DRG neuron culture	NDEVs	TRPV1 activation promoted the release of miR-21-5p and miR-23a in NDEVs	Activated M1 macrophages	[53, 54]
2020	SNI	Serum-derived sEVs	Increased serum sEVs contained C5a and ICAM-1	/	[101]
2020	Metastatic cancer pain	Cancer cell-derivd sEVs	Increased gene expressions in sEVs	Induced mechanical allodynia and thermal hyperalgesia	[106]
2017	Lumbar disc herniation	NPs	Increased miR-223 in NPEVs at the acute phase	Attenuated the neuronal activity in the pain pathways	[114]
2017	CFA	Endosomes	Activated NK1R and CLR on endosomes	Promoted pain transmission	[121, 122]
2016	SNL	MDEVs	Increased MDEV-IL-1β in CSF and spinal cord	Reduced PWT and PWL	[59]

*NDEV *Neuron-derived extracellular vesicle, *SNI *Spared nerve injury, *SNL *Spinal nerve ligation, *MDEV *Microglial-derived extracellular vesicles, *DRG *Dorsal root ganglion, *PWT *Paw withdrawal threshold, *PWL *Paw withdrawal latency, *CIBP *Cancer-induced bone pain, *NP *Nucleus pulposus, *CFA *Complete Freund’s adjuvant, *TRPV-1 *Transient receptor potential vanilloid 1, *CLR *Calcitonin receptor-like receptorsm *NK1R *Neurokinin 1 receptor, *OPRM1 *µ1 opioid receptor, *DRG *Dorsal root ganglion

#### Neuron-derived sEVs

Changes in synaptic plasticity contribute significantly to the occurrence and development of chronic pain. The roles of neuron-derived sEVs (NDEVs) in synaptic signal transmission have been widely explored. It was demonstrated that MVBs, which are responsible for the biogenesis of sEVs, were present in the axons, terminals of neurons, soma, and dendrites [45]. MVBs were found to be 50 times more abundant in the soma and dendrites than in the axons. Another research reported that neuronal CD63-GFP^+^ ILVs in MVBs were primarily localized in soma and dendrites, but not in axonal terminals in vitro and in vivo by confocal image analysis [46], potentially emphasizing the production of MVBs in soma and transportation to axons and terminal before sEVs release. Moreover, these MVBs exhibited bidirectional transport capabilities, enabling them to move in different directions, including from axon to terminal and potential anterograde direction [45]. Such intricate transportation mechanisms contribute significantly to the physiological functions of neuronal sEVs.

The irregular secretion of synaptic sEVs by neurons has been associated with the development of pain disorders by disrupting the proper transmission of synaptic signals. According to observations, glutamatergic activity promoted the release of NDEVs [47]. The released sEVs carried glutamate receptor-2 (GluR2) subunits, which resulted in the loss of AMPA receptors in synapses. The presence of GluR2 subunits in NDEVs, coupled with the increased secretion of sEVs following glutamatergic synaptic activation, highlighted sEV release as a plausible method for local receptor elimination at synapses undergoing plastic changes [47]. It was demonstrated that the involvement of glutamatergic synapses has been in the processes underlying pain sensation [48]. EVs released in the synapse cleft may actively participate in the physiological and pathological processes of chronic pain in this way. Long-term potentiation (LTP), a critical cellular mechanism underlying chronic pain, is characterized by enhanced synaptic transmission due to persistent changes in synaptic efficacy and plasticity. Notably, an increase in the release of neurotransmitters encapsulated in presynaptic vesicles has been observed during LTP induction. Two hours after LTP, there was also an increase in the density of vesicles closely associated with the synaptic membrane. These structural changes play a vital role in the sustained enhancement of neurotransmitter release probability observed following LTP induction [49]. It has been demonstrated that sEVs were capable of transmitting synaptotagmin 4 (Syt4), a protein involved in retrograde signaling, to postsynaptic cells [50]. Retrograde signaling originating from postsynaptic targets is critical for maintaining synaptic plasticity. In addition to the number of sEVs released in the synaptic cleft affecting neuronal function, their contents also influence synaptic plasticity. In particular, transmembrane protein proline-rich 7 (PRR7) was activity-dependently released by neurons *via* sEVs. sEV-PRR7 was taken up by neurons through membrane fusion and eliminated excitatory synapses but not inhibitory synapse numbers in local neurons [51].

Apart from their role in the synaptic cleft, emerging evidence suggests that NDEVs are capable of modulating the function of surrounding cells during painful states. Specifically, NDEV-mediated communication between neurons and other cells, namely glia and macrophages, has been shown to play a crucial role in both pain development and various neurological disorders. A substantial of NDEVs presenced in the perineuronal space after nerve injury, with their origin being synapses, and were subsequently transferred to microglia. They also significantly contributed to the recruitment and activation of microglial cells [52]. TRPV-1, an ion channel known for its involvement in pain perception, has been identified as an important player in NDEVs production. Activation of TRPV-1 receptors has been found to promote the release of primary sensory NDEVs carrying specific microRNAs (miRNAs), such as miR-21-5p [53]. Conditional deletion of miR-21-5p in sensory neurons was shown to reduce hypersensitivity and inflammatory macrophage recruitment in the dorsal root ganglion [53]. Similarly, after nerve injury, miR-23a was upregulated in NDEVs. The NDEV-enriched miR-23a was demonstrated to exacerbate neuropathic pain by promoting M1 polarization upon uptake by macrophages [54]. Furthermore, NDEVs have been found to exert neuroprotective effects on microglia via inhibition of apoptosis and inflammation [55]. Hypoxia preconditioning has been shown to alter the content of NDEVs, inducing increased miR-126-3p expression in sEVs. Notably, miR-126-3p-enriched NDEVs were found to modulate inflammatory signaling pathways, ultimately mitigating ischemia-reperfusion-induced pain [56]. In addition to their potential therapeutic applications, NDEVs have also been implicated in functional behavioral recovery following SCI. The NDEV-miR-124-3p was observed to be potentially internalized into microglia and astrocytes [46, 57]. It mitigated the activation of microglia and astrocytes, thereby facilitating recovery from SCI [57]. Furthermore, NDEV-miR-124-3p was found to enhance the expression of glutamate transporter-1 (GLT1) by repressing miR-132 and miR-218 which hinder GLT1 in astrocytes [46, 58]. GLT1 presented on astrocytes participates in maintaining the balance of synaptic glutamate in tripartite synapses.

**Fig. 2:**
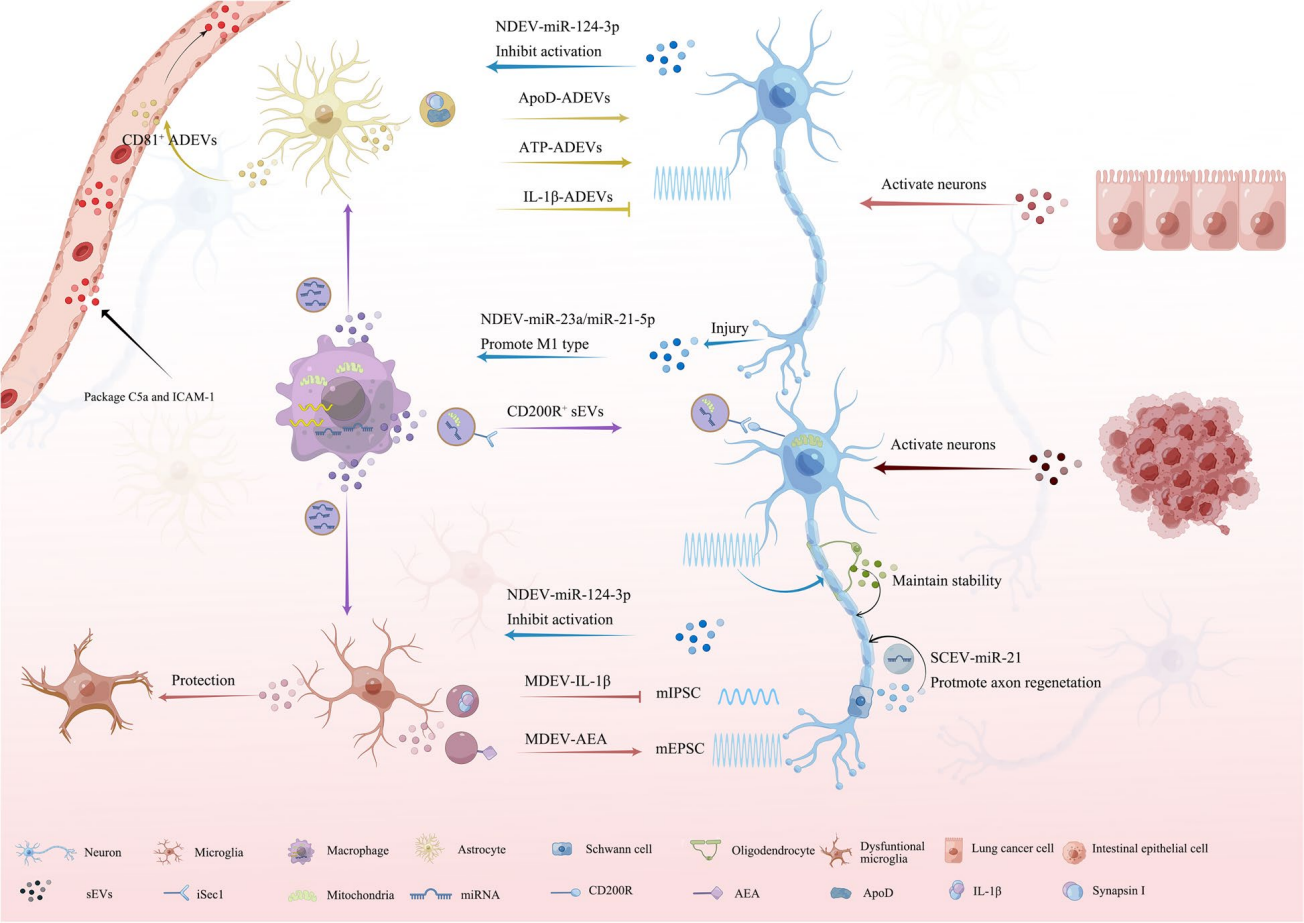
sEVs in pain signal transmission. SEVs are released by various cells within the nervous and immune systems. These vesicles transport miRNAs, proteins, or lipids to target cells, facilitating signal transmission during the pain process. sEV: small extracellular vesicle

**Fig. 3:**
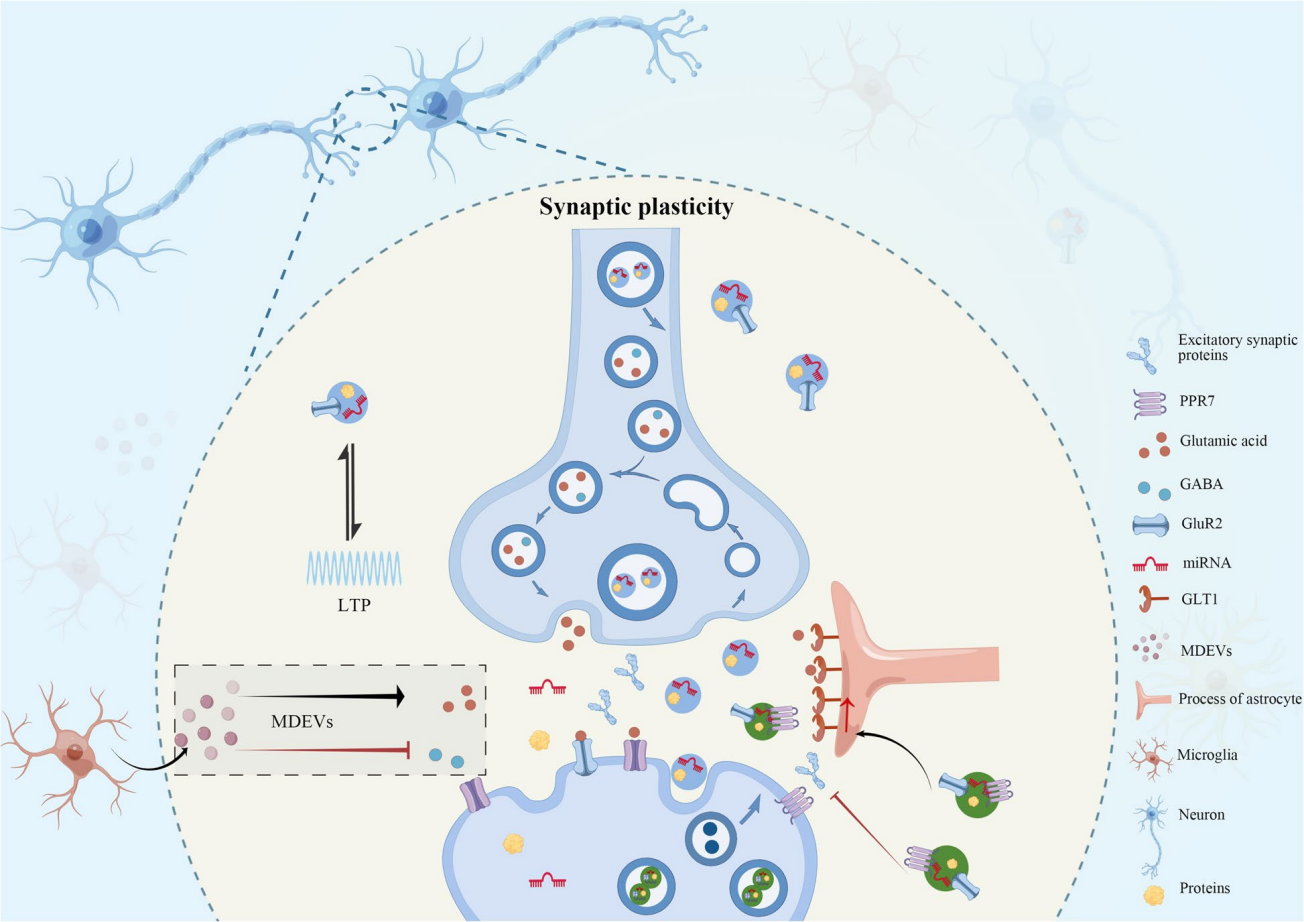
sEVs in synaptic plasticity. The sEVs encapsulate neurotransmitters, membrane proteins, RNAs, receptors et al. NDEVs promote the expressions of GLT1 on astrocytes and carry out GluR2 to reduce AMPA receptors in synapses. These effects balance glutamate activity in the synaptic cleft. Moreover, LTP promotes the production of NDEVs, and the released synaptic vesicles participate in the formation of LTP. MDEVs promote the excitation of neurons by enhancing glutamatergic transmission and inhibiting GABA transmission. NDEV-PRR7 eliminates excitatory synapse proteins. sEV: small extracellular vesicle; GluR2: glutamate receptor-2; GLT1: glutamate transporter-1; NDEVs: neuron-derived small extracellular vesicles; MDEV: microglia-derived small extracellular vesicle; LTP: long-term potentiation; PRR7: Proline-rich 7; GABA: γ-aminobutyric acid

 Overall, these findings shed light on the complex interplay between NDEVs and surrounding cells, highlighting them as a communication mediator in pain states (Figs. 2 and 3). Given the difficulties in isolating sEVs derived from neurons in vivo, many studies have resorted to ex vivo culture systems for sEV isolation. As increasingly sophisticated techniques are developed, future research should aim to investigate the functions of sEVs produced within the nervous system in vivo.

#### Microglia-derived sEVs

Microglia are the resident macrophages of the central nervous system (CNS) and spinal cord, acting as sentinels to external insults. Recent studies have shed light on the function of microglia-derived sEVs (MDEVs) in neuroinflammatory responses and pain modulation. The heterogeneous origins and complex environmental influences have a noteworthy impact on the function of MDEVs within the nervous system. Whether these effects are beneficial or detrimental remains a subject of intense investigation.

In the context of spinal nerve ligation (SNL)-induced pain, MDEVs have been found to significantly increase in both the cerebrospinal fluid (CSF) and dorsal horn of the spinal cord. The process of MDEV shedding was regulated by the activated P2 X 7-p38 signaling pathway in this model [59]. Interestingly, exposure to these sEVs has been found to elicit considerable increases in spontaneous excitatory postsynaptic current (sEPSC) frequency and amplitude, regardless of SNL presence [59]. Notably, interleukin-1 beta (IL-1β), packaged within these vesicles, has been found to partially mediate the pain effects of MDEVs [59]. Furthermore, MDEVs induced alteration of excitation/inhibition balance. The studies have demonstrated that normal microglia-derived sEVs enhanced glutamatergic transmission through the promotion of ceramide and sphingosine synthesis in excitatory neurons, which was essential for modulating synaptic activity [59–61]. On the other hand, they modulated the inhibitory neurons. It indicated an association between the generation of MDEVs and the release of endocannabinoids (eCBs) [61, 62], which was known to modulate nociceptive function in all phases of pain processing pathways [63]. Specifically, MDEVs were capable of transporting N-arachidonoylethanolamine (AEA), a type of eCB, on their membrane. MDEV-AEA activated presynaptic type-1 cannabinoid receptors (CB1) on neurons and suppressed the spontaneous release of γ-aminobutyric acid (GABA), inducing a significant decrease in miniature inhibitory postsynaptic currents (mIPSCs) frequency but not amplitude [61]. The augmented glutamatergic transmission and diminished GABAergic transmission mediated by MDEVs imply that the escalated release of MEDVs after nerve injury may serve as a mechanism through which microglia contribute to excitatory phenomena in the context of pain processing (Fig. 3). Intriguingly, sEVs isolated from microglia in medicinal leeches’ central nervous system exhibited neuroprotective properties by augmenting neurite outgrowth in rat neurons [64]. Such intercellular communication and sEV-mediated signaling may be critical for the effective functioning of the nervous system across species.

MDEVs exhibit substantial potential as intercellular communicators and their regulatory influence over their parent cellular population have been established (Fig. 2). Notably, sEVs isolated from inflammatory microglia have been found to modulate the gene expression profile of recipient microglia. RNA sequencing data confirmed that the intake of sEVs originating from normal microglia or 10 ng/ml TNF-α-prepared microglia led to a notable decrease in gene expression levels linked with inflammasome activation, neuroinflammation, and apoptosis signaling in different microglia types [65]. Furthermore, in vitro-generated sEVs derived from both non-polarized and polarized microglia could transfer a protective phenotype to dysfunctional microglia [65, 66]. These studies have explored the function of MDEVs in vitro and suggested that they possessed a protective effect on their parent population. The potential impact of sEVs derived from distinct microglial states on the modulation of pain remains uncharted territory. Moreover, proinflammatory microglia produced various miRNAs and cytokines that contributed to the promotion of neuroinflammation. Several miRNAs (miR-375, miR-146a-5p, miR-181a, miR-223, miR-155) [67–69] and inflammatory mediators (IL-1β, TNF-α, IL-6) [70, 71] were identified within the sEVs originating from M1 microglia, thereby contributing to neuroinflammation. These biomolecules contribute to neuroinflammation or nerve injury. Studying MDEVs in vivo poses challenges, thus most current research relies on ex vivo cell culture to obtain these vesicles. Furthermore, most published studies have focused on investigating the functional effects of sEVs derived from different microglial subtypes on neuroinflammation and nerve injury, but investigations into the underlying mechanisms remain incomplete.

#### Astrocyte-derived sEVs

Astrocytes played a crucial role in the pathophysiology of pain disorders, as they altered the microenvironment that surrounded neurons [43] (Fig. 2). ADEVs carried fibulin-2 on the surface of sEVs, and the activation of TGF-β signaling mediated by ADEV-fibulin-2 promoted the development of dendritic spines and synapses [72]. The recent research has demonstrated that ADEVs carrying apolipoprotein D (ApoD), a protein known for its neuroprotective properties, can be effectively conveyed to neurons. This transfer was found to confer resistance to oxidative stress [73]. Experiments involving ApoD-knockout ADEVs demonstrated partial protective effects, whereas ApoD-positive ADEVs provided full neuroprotection and enhanced neuronal survival [73]. Moreover, ADEVs containing synapsin I, which banded to neural cell adhesion molecules (NCAM) and promoted neurite outgrowth, synaptic plasticity, and neuronal survival [74], exerted a protective effect on neurons. The functional state of astrocytes influenced the ability of ADEVs to modulate neuronal excitability and maintain homeostasis under various microenvironmental stimuli [75]. SEVs from ATP or the anti-inflammatory cytokine interleukin-10 (IL-10)-primed astrocytes, could promote dendritic branching, regulate synaptic transmission, and increase neuronal survival [76]. Conversely, ADEVs secreted under the influence of pro-inflammatory cytokines, such as IL-1β or TNF-α, modulated peripheral immune response and promoted immune cell trafficking into the central nervous system [76]. Exposure to ADEV-ATP has been shown to induce heightened spike and burst activities in neuronal cultures, while exposure to ADEV-IL-1β led to decreased spike and burst activity in the same model [76]. Thus, sEVs derived from astrocytes are capable of modulating synapse transmission and influencing neuronal activity depending on the condition of parent cells.

A recent study has established that the efficacy of analgesic medicine is closely linked to ADEVs, especially opiate abuse. The upregulation of sonic hedgehog signaling molecules on these vesicles has been found to induce morphine tolerance, thereby reducing the effectiveness of analgesic drugs. Further research has demonstrated that inhibiting the release of ADEVs could delay the onset of morphine tolerance in mice [77]. Moreover, ADEVs containing miR-138 were discovered once exposed to morphine. Upon being internalized by microglia, these sEVs activated the microglia and provoked neuroinflammation [78]. It is therefore inferred that in vivo production of ADEVs may be a crucial factor contributing to the tolerance of analgesic drugs, and therefore affecting their therapeutic efficacy.

#### Schwann cell-derived sEVs

Reprogramming differentiated Schwann cells (dSCs) into repair Schwann cells (rSCs) is a crucial factor in restoring functionality following peripheral nerve injury. This reprogramming process promoted axonal regeneration and tissue homeostasis, as has been reported previously [79, 80]. Interestingly, axons specifically internalize sEVs released by rSCs (SDEVs), further highlighting the importance of these reparative cells in the regenerative process [80]. Notably, heightened EV-miR-21 expression in rSCs was found to be responsible for their pro-regenerative capacity. Specifically, the downregulation of PTEN and PI3-kinase activation in neurons was linked to the effect of SDEV-miR-21 after nerve injury [81] (Fig. 2). Moreover, it was reported that after mechanical stimulation of Schwann cells, the SDEVs contained much more miR-23b-3p. The miR-23b-3p in SDEVs targeted neuropilin 1 in neurons to promote peripheral nerve injury repair [79]. It is worth noting that proteins in SDEVs may also have a significant impact on the process. The proteomic analysis of SDEVs revealed that twelve proteins, including lotillin-2, neuropilin-2, septin-7, and syntenin-1 et al., exhibited close associations with axon regeneration [82]. SDEVs also inhibit inflammation in neuroinflammation [82, 83]. These findings underscore the therapeutic potential of rSCs-derived sEVs in promoting peripheral nerve repair and recovery. Some issues limit the utilization of these sEVs. The culture conditions of rSCs play a critical role in achieving the optimal efficacy of sEVs [83]. Additionally, a commonly faced problem in utilizing sEVs arises from the challenges related to isolating and addressing their heterogeneity. Furthermore, due to technological limitations, sEVs cannot be manufactured in substantial quantities.

#### Satellite glia-derived sEVs

In ganglia, satellite glial cells (SGCs) envelop sensory neurons nearby, ensuring neuronal homeostasis. Upon nerve injury or inflammation, SGCs could activate and elicit the upregulation of ion channels, gap junctions, and receptors on their surface, thereby triggering neuronal excitation and pain development [84]. In a recent report, SGCs were found to release sEVs, which exhibited a modified protein profile under inflammation stimulation [85], suggesting a potential role of sEV-contained proteins in inflammatory pain conditions. Despite these breakthrough discoveries, the precise influence of SGC-EVs on neuronal excitability and pain genesis requires further exploration.

#### Oligodendrocyte-derived sEVs

Oligodendrocytes, the myelin-producing cells in CNS, have long been known for their role in insulating and protecting neuronal axons, ensuring efficient nerve conduction. According to recent research, oligodendrocyte function has been found to have an additional dimension involving the release of sEVs that contain a variety of bioactive molecules. These sEVs were absorbed by neurons and played a crucial role in enhancing their metabolic health, facilitating axonal transport, and maintaining their structural integrity even under stressful conditions [86] (Fig. 2). The secretion of oligodendroglial-derived sEVs was initiated through the release of the neurotransmitter glutamate in an activity-dependent manner. This process was facilitated by the influx of Ca^2+^ *via* oligodendroglial N-methyl-D-aspartate receptor (NMDA) and AMPA receptors [30]. Once released, these sEVs are internalized by neurons, contributing to their maintenance [30]. Dysfunction of oligodendrocyte has been linked to abnormal neuronal conduction and implicated in the pathogenesis of neurological disorders, such as spinal cord injury.

### The sEVs of the periphery origin

#### Macrophage-derived sEVs

The significant role of macrophages in pain initiation and progression has been widely acknowledged [87, 88]. SEVs originating from these immune cells have emerged as key mediators of intercellular communication between peripheral and central tissues. Recent investigations have uncovered that sEVs derived from various macrophage subsets depicted diverse effects on the nociceptive process (Fig. 2).

Delayed resolution of inflammation in acute inflammatory pain may contribute to the development of chronic pain. Recent studies have shown that M2-type macrophages, with an increase in the CD206^+^ M2-like marker observed during pain resolution, play a critical role in resolving inflammatory pain. Conversely, treatment with M1 macrophages induced transient hyperalgesia in normal mice and did not affect resolving inflammatory hyperalgesia in models of inflammatory pain [21]. sEVs derived from M2 macrophages have been shown to mediate the communication between immune and neuronal systems, highlighting their potential as analgesic agents during the resolution of inflammatory pain. It was found that CD206^+^ M2-like macrophage-derived sEVs rapidly resolved transient inflammatory pain during the subsiding phase [21]. Furthermore, the presence of mitochondria in sEVs derived from M2 macrophages was found to play a critical role in the analgesic process. Mitochondrial dysfunction and defects have been implicated in the pathogenesis of various chronic pain conditions [89–91]. The transplantation of mitochondria to neurons will sustain the neuronal function in a pathological condition [92]. These macrophages were capable of transferring mitochondria to neurons via sEVs, thereby maintaining a functional mitochondrial network in sensory neurons [21, 93]. Ligand-receptor interactions have been reported to specifically target neurons by sEVs. CD200R^+^ sEVs were found to target iSec1^+^ neurons for mitochondria delivery, and in vivo studies have confirmed the necessity of CD200R expression on macrophages and iSec1 expression on sensory neurons for effective transport of mitochondria by sEVs during the resolution of inflammatory pain [21]. In this study, the peak size of sEVs was around 100 nm. However, it is worth noting that mitochondria typically have a diameter greater than 200 nm. Interestingly, it was reported that MVs but not exosomes transfer mitochondria to neurons’ brain slices [93]. Therefore, vesicles may be carrying and transporting mitochondrial DNA (mtDNA), RNA (mtRNA) [94], or mitochondrial bodies [95]. The transfer of such material may promote inflammatory pain resolution rather than the mitochondria themselves. Further investigation is needed to explore this hypothesis.

Based on well-established findings, diverse subpopulations of macrophages have been shown to induce analgesic responses to a certain extent (Table 3). It has been reported that naïve macrophages (M0) could reverse thermal hyperalgesia caused by Complete Freund’s Adjuvant (CFA) injection after 48 h, but they were unable to relieve mechanical hypersensitivity [96]. Mitochondria-containing sEVs isolated from M2-type macrophages have been shown to rapidly and transiently resolve inflammatory pain, while artificial vesicles containing mitochondria from M0 macrophage cell bodies also alleviated inflammatory pain [21]. In addition to promoting pain self-regression, M2 macrophage-secreted sEVs can also serve as effective vectors for delivering nucleic acids. SEVs carrying miR-23a-3p from macrophages were found to elevate the mechanical allodynia and thermal hyperalgesia thresholds in an inflammatory pain model [97]. This effect was attributed to the efficient transport of miR-23a-3p to microglia by these sEVs [97]. Besides the analgesic effect of sEVs from M0 macrophages, M1- and M2-type macrophage-derived sEVs also have analgesic effects. It is well established that lipopolysaccharide (LPS) stimulation effectively triggers macrophage polarization towards the classically activated M1 subtype. SEVs were obtained after the stimulation of RAW 264.7 cells with 1 µg/ml LPS for a duration of 24 h. These sEVs exhibited noteworthy attenuation of thermal hyperalgesia but no discernible impact on mechanical allodynia in a CFA-induced inflammatory pain model as early as 24 h post-administration [96]. For longer analgesic effects, the administration of sEVs only reversed thermal hyperalgesia after 48 h of CFA injection [96]. Moreover, sEVs derived from LPS-primed macrophages carrying anti-inflammatory microRNAs (miR-146a, miR-146b, and miR-21) have been found to transfer to neurons, astrocytes, and microglial cells. Intrathecal administration of sEVs reduced mechanical hypersensitivity in formalin-induced late-phase inflammatory pain, and prophylactic administration of sEVs two weeks before induction of pain was effective in attenuating pain hypersensitivity in the CFA model [98]. According to these results, sEVs from different subtypes of macrophages all have varying degrees of analgesic effects.

**Table 3:** The effect of sEVs on pain relief

Published year	Model	Resources	sEVs method	Treatment method	Dose	Effects	Speculated mechanisms	Ref.
2023	CCI	huc-MSCs	Sequential centrifugation	i.t.	5 µg on 2, 4, and 6 days after CCI surgery	Ameliorated mechanical allodynia and inhibited microglia	Encapsulated miR-99b-3p promoted microglial autophagy and inhibited inflammation	[145]
2023	CCI	huc-MSCs	Sequential centrifugation	i.t.	5 µg on days 2, 4, and 6 after surgery	Ameliorated mechanical allodynia; inhibited microglial activation and restrained activation of the TLR2/MyD88/NF-κB signal	/	[143]
2022	Carrageenan-induced pain	CD206^+^ M2 macrophages	Gradient centrifugation	i.t.	5 µl (100 µl sEVs from 10 × 10^7^)	/	sEVs transferred mitochondria to sensory neurons	[21]
2022	SNI	hPMSCs	Gradient centrifugation	i.t.	5 µg	Alleviated hind paw withdrawal threshold	Encapsulated miR-26a-5p regulated Wnt5a/Ryk/CaMKII/NFAT signal	[144]
2022	Tendinopathy	iMSCs	Gradient centrifugation	Local injection	1 × 10^9^ particles/ml once a week for 4 weeks	Alleviated hind paw withdrawal threshold; inhibited the activation of mast cells	/	[137]
2021	CFA	M1-type macrophages	Gradient centrifugation	i.t.	1 µg, a single injection	Attenuated mechanical hyperalgesia	Anti-inflammatory miRNAs	[98]
2021	Formalin model	M0- and M1-type macrophages	Gradient centrifugation	i.t.	1 µg, 48 h before formalin injection	Reduced the late-phase formalin-induced nociceptive response and spontaneous pain	/	[98]
2021	IR-pain model	Neurons	Gradient centrifugation	i.t.	20 µg in 20 µl	Improved mechanical sensitivity and thermal allodynia	miR-126-3p-enriched Hypo-VSC sEVs modulated PIK3R2-mediated PI3K/Akt and NF-κB signaling pathways	[56]
2021	EPA	iMSCs	Gradient centrifugation	i.v.	1 × 10^10^ particles weekly	Reversed pain behavior; reduced the overexpression of COX-2	/	[146]
2020	OA	Rat BMSCs	Ultracentrifugation	Local injection	40 µg/100 µl	Relieved pain; promoted cartilage repair and extracellular matrix synthesis	/	[138]
2020	CFA	RAW264.7	Gradient centrifugation	Local injection	20 µl (0.5 µg)	Increased thermal pain and mechanical pain thresholds	sEVs containing miR-23a-3p regulated HDAC2/NRF2 axis by decreasing USP5 expression	[97]
2020	SNL	huc-MSCs	Ultrafiltration	Local treatment	Wrapped around nerve	Improved withdrawal threshold and latency; ameliorated inflammatory microenvironment	/	[172]
2020	DNP	Mouse BMSCs	Gradient centrifugation	i.v.	1 × 10^9^ particles weekly for 8 weeks	Improved neurological outcomes; downregulated TLR4/NF-κB signaling pathway in the sciatic nerve tissues	/	[136]
2019	SNL	huc-MSCs	Ultrafiltration	i.t.	Single 1.2 mg/ml or continuous infusion (hourly dose: 1.2 mg)	Attenuated mechanical allodynia and thermal hyperalgesia; inhibited neuron and glial activation and inflammation	/	[135]
2014	CFA	RAW264.7	Gradient centrifugation	Local injection	20 µl (0.5 µg)	Relieve thermal hyperalgesia and paw swelling	/	[96]
2005	Collagen-induced arthritis	Dendritic cells	Gradient centrifugation	i.v.	1 µg in 10 µl	Reduced paw swelling	/	[185]

*huc-MSCs *human umbilical cord mesenchymal stem cells, *hPMSCs *human placental mesenchymal stem cells, *iMSCs *induced pluripotent stem cell-derived MSCs, *BMSCs *Bone marrow mesenchymal stem cell, *DNP *Diabetic peripheral neuropathy, *EPA *Experimental autoimmune prostatitis, *SNI *Spared nerve injury, *SNL *Spinal nerve ligation, *CCI *Chronic constriction injury, *CFA *Complete Freund’s adjuvant

#### Blood-derived sEVs

Accumulating evidence has suggested that sEVs hold immense potential for diagnostic and prognostic purposes across multiple disorders [99, 100]. In the context of nerve injury and neuroinflammation, alterations in sEV composition, markers, and abundance have been observed in serum. Proteomics analysis of serum sEVs in a spared nerve injury model of neuropathic pain revealed significant differences between SNI and control samples. Notably, complement component 5a (C5a) and intercellular adhesion molecule-1 (ICAM-1) were found to be increased within sEVs from the serum of neuropathic pain, whereas they were decreased in the whole serum of neuropathic pain compared to controls [101]. This suggested that rather than an increase in production, neuropathic pain might induce the packaging of C5a and ICAM-1 protein into sEVs, thereby indicating the potential value of altered sEV content as a diagnostic tool [101]. Complex Regional Pain Syndrome (CRPS) is a neuropathic pain disorder that is known for its refractory nature. In a recent study, researchers revealed significant differences in the levels of 17 miRNAs of sEVs between responders and poor responders before and after the plasma exchange therapy. Among these study participants, individuals who exhibited a poor response had reduced levels of sEV-hsa-miR-338-5p. This miRNA targets interleukin 6 (IL-6) and modulates IL-6 mRNA and protein expression levels [102]. In addition, the researchers employed miRNA-based markers to distinguish the heterogeneous patient population with CRPS, which was considered crucial for identifying suitable treatment options. After obtaining serum samples from various species, the serum was subjected to a 24-hour incubation period at a temperature of 37 °C. It was observed that the conditioned serum exhibited analgesic properties toward neuropathic pain *via* sEVs [103]. The serum with sEVs was found to contain higher levels of anti-inflammatory and pro-resolving mediators, including IL-1Ra, Tissue Inhibitor of Metalloproteinases 1 (TIMP-1), and TGF-β1, as well as resolvins D1/D2 [103]. Flow cytometry represents a potential solution for distinguishing markers present on sEVs derived from parent cells, enabling differentiation of the various sources of these sEVs.

#### Tumor-derived sEVs

Tumor-derived sEVs possess the ability to facilitate perineural invasion and modulate nociceptive response, thereby playing a crucial role in cancer pain. Administering tumor-derived sEVs through intraplantar injection in naive mice resulted in heightened sensitivity by sensitizing C-fiber nociceptors [104]. In pancreatic cancer, these tumor-derived sEVs were taken up by dorsal root ganglion (DRG) neurons, triggering the expression of nociceptor genes [105]. Furthermore, the enriched-miR-21-5p molecule was implicated in contributing to the overall impact of these sEVs on pancreatic cancer pain [105]. The tumor-derived sEVs also led to hypersensitivity through an autotaxin (ATX)-enzyme synthetizing lysophosphatidic acid (LPA)-LPA receptor (LPAR)-dependent manner in bone cancer pain [104]. In vitro, studies also revealed tumor-sEVs evoked dorsal root ganglion (DRG) neuron sensitization by ATX-LPA-LPAR signaling [104]. Remarkably, depleting sEVs from an oral cancer cell line supernatant led to reduced mechanical allodynia and eliminated thermal hyperalgesia in vivo [106, 107]. The miR-21-5p and miR-221-3p in sEVs possibly contributed to oral cancer pain [107]. Similarly, the other biomolecules that tumor cell-derived sEVs carried like nerve growth factor receptor cytokines are able to promote immune cell aggregation and lead to neuron sensitization [108, 109]. Furthermore, upon uptake of these sEVs by target cells, an exacerbation of tissue damage was induced through the active vision of intracellular signaling pathways such as mitogen-activated protein kinase (MAPK) and nuclear factor κ B (NF-κB) [108].

Intercellular communication by sEVs is a key feature of metastasis. They transport bioactive molecules associated with tumor progression. Cancer-induced bone pain (CIBP) is a typical cancer pain caused by metastasis. It is a pervasive issue that results from the metastasis of malignant tumors to the bone. CIBP constitutes over 50% of chronic cancer pain cases [110]. Recent studies have provided insights into the influence of sEVs on CIBP. SEVs derived from lung cancer cells were found to exacerbate pain behavior in mice with CIBP [111]. These sEVs carried let-7d-5p, which inhibited the µ1 opioid receptor (OPRM1) in the DRG, thereby contributing to the heightened pain effects induced by sEVs. It was identified 40 genes were overexpressed in metastatic tumors from individuals reporting high levels of pain compared to N0 tumors and normal tissue. These genes have both oncogenic and neuronal functions and were presented in sEVs. Moreover, cancer cells generally produce more sEVs than normal cells, and sEVs derived from cancer cells have a strong capacity to modify both local and distant microenvironments [112, 113]. These findings offer valuable insights into the role of sEVs in cancer pain and highlight the potential therapeutic benefits of targeting sEVs and associated gene expression in the management of this debilitating condition.

#### Other sources-derived sEVs

Intervertebral disc (IVD) degeneration is a prevalent precursor of low back pain, a condition that affects millions worldwide. The herniation of IVDs also results in heightened nociceptive responses in the spinal cord, ultimately leading to radicular pain upon exposure of nerve roots to nucleus pulposus (NP) tissues. Recent research has demonstrated that sEVs released by NP cells have an impact on persistent pain after disc herniation. In particular, miR-223 found in NP-derived sEVs (NPEVs) was shown to have a potential anti-nociceptive role during the acute phase of disc herniation [114]. The increased expression of miR-223 during this phase was found to be associated with a decreased risk of radicular pain [114]. Additionally, the process of reprogramming NP cells utilizing Forkhead box F1 (FOXF1) resulted in the delivery of sEVs containing FOXF1 into the IVD. Consequently, this intervention provided a state that was both anti-catabolic and anti-inflammatory, which helped to ameliorate the condition of IVD degeneration [115].

Visceral pain is a distressing sensation that results from the activation of nociceptors located in internal organs or tissues, such as IBD or endometriosis. A growing body of evidence suggests that the gut and nervous system are intricately connected through a network of interconnections known as the gut-brain axis. Alongside the well-known secretion of neurotransmitters, metabolites, and pathogen-associated molecular patterns (PAMPs), emerging research has uncovered the release of sEVs by gut microbiota and intestinal epithelial cells. These sEVs could interact with local neuronal and immune cells in the gut [116, 117]. These interactions can modulate signal transmission in the vagal afferent pathways, shedding light on the important regulatory role of the released sEVs in host physiology and pathology. It was identified that miRNA-6769-5p regulated the target gene Ataxin 1 (ATXN1), which was involved in intestinal sEV-triggered activation of neuronal cells [118]. The disruption of gut microbiota led to the release of sEVs from intestinal epithelial cells, which caused M1 polarization in mesenteric lymph nodes and resulted in an increased level of circulating IL-1β. The elevated level of IL-1β promoted neuronal damage and apoptosis [119]. On the other hand, the nervous system can also regulate intestinal function in reverse. Recent literature has reported that gut-innervating TRPV1^+^ nociceptors regulated the composition of the intestinal microbiota to limit inflammation and promote intestinal tissue protection [120]. These evidences suggest that sEVs may play a role in regulation among the epithelial cells, microbiota, and nervous system. Given their ability to modulate neural activity, it is likely that sEVs contribute to both the development and resolution of visceral pain.

As mentioned above, the intracellular precursors of exosomes are formed in the endosomal pathway. Endosomes are vesicular structures present within cells before the formation of exosomes. Interestingly, they have also emerged as significant players in the dynamic intercellular communication network. While the majority of studies highlight the diverse alterations arising from cell surface receptor signaling, a cohort of scientists have unearthed a notable function of the inner endosomes in pain modulation. Specifically, it has been unveiled that endosomes, *via* their surface-expressed GPCRs such as calcitonin receptor-like receptors (CLRs) and neurokinin 1 receptor (NK1R), actively contributed to the pathophysiology of pain [121–123]. The utilization of nanoparticles that targeted GPCRs on endosomes resulted in profound and sustained alleviation of nociceptive, inflammatory, and neuropathic pain [124]. It is worth noting that the endosomes carrying these GPCRs have the potential to undergo a process of exosome formation, whereby they release them into the extracellular milieu. These results provide insights into the significance of endosomes and the process of sEV formation in alleviating pain.

### sEVs as biomarkers

Clinical pain disorders are characterized by the presence of persistent pain, which is a subjective sensory experience. This poses challenges for objective assessment, as doctors face difficulties in distinguishing between emotional features and somatic sensations using scoring systems. Nevertheless, there is potential for diagnosis through biomarkers associated with underlying mechanisms, neural activity, and susceptibility [125]. Neuroimaging can provide real-time image biomarkers [126], but biological markers are better suited to assess the underlying pathological mechanisms. The use of sEVs as biomarkers has been extensively investigated across various disorders, with the development of a specific commercial test kit for prostate cancer [127]. SEVs, as biomarkers, have been extensively studied in numerous disorders due to their distinctive advantages. Firstly, sEVs can be readily obtained from various liquids. Secondly, they possess the capability to traverse both the BBB and the blood-spinal cord barrier BSB, enabling them to transport biomolecules originating from the nervous system. Thirdly, sEVs encapsulate a multitude of essential components such as proteins, DNA, RNA, lipids, and others, derived from their parent cells, thereby offering valuable insights into pathological tissues. Lastly, aberrant expression of certain miRNAs and proteins has been linked to the development and maintenance of pain conditions. These molecules can modulate key pathways and mechanisms involved in pain perception. By targeting specific genes involved in these processes, miRNAs and proteins can influence the overall pain response. The detection of miRNAs and proteins in sEVs offers a unique opportunity for biomarker discovery in pain research. SEVs are membrane-bound structures that cells release into the extracellular space. One of their primary functions is to protect enclosed molecules from degradation, making them valuable for identifying novel biomarkers within SEVs [127].

In mice with SNL-induced neuropathic pain, there was a significant increase in the levels of miR-21 in both DRG and blood-derived sEVs [128]. Additionally, miR-23a levels in sEVs derived from DRG neurons were found to increase after peripheral nerve injury, suggesting that contained-miR-23a sEVs could serve as a prospective biomarker for pain hypersensitivity [54]. These findings suggest that sEV-miRNAs from blood or injured sites have the potential as biomarkers for neuropathic pain induced by nerve injury. Recent research also found that following SCI, there were notable changes in circulatory CD81^+^ sEVs expressing, as well as their associated miRNAs cargo. These acute alterations hold the potential to elicit an inflammatory response in the brain cortex through the circulation, with astrocytes appearing to play a pivotal role in both the source and response to circulating sEVs post-injury [129]. Circulatory CD81^+^ sEVs exhibit a potential role in characterizing the pain that results from SCI. The increased expression of sEV-miR-223 from NPs during the acute phase of disc herniation was found to be associated with a decreased risk of radicular pain [114]. SEVs also exhibit potential as prognostic indicators for pain management, according to recent research findings. In particular, following therapeutic plasma exchange treatment for CRPS, significantly lower levels of sEV-miR-338-5p were observed in blood samples from poor responders [130]. Analysis of sEV-miR-338-5p may therefore represent a viable method for evaluating the clinical efficacy of plasma exchange treatment.

To shed light on the potential of sEVs as reliable biomarkers for pain diagnosis, further investigations are warranted. Specifically, a comprehensive examination of the role of sEVs derived from nerve roots, brain, CSF, and serum in various pain conditions is required. Such investigations would not only allow for a deeper understanding of the involvement of sEVs in pain disorders but also pave the way for the development of effective pain management strategies. The unique properties of sEVs, including their ability to carry specific cargoes such as proteins, lipids, and nucleic acids, make them attractive candidates for use as diagnostic tools. By elucidating the specific molecular contents of sEVs associated with different types of pain, it may be possible to identify objective biomarkers that can be used to diagnose pain conditions and monitor treatment efficacy. However, several technological hurdles must be overcome to establish the reliable diagnosis based on sEVs: (1) the different isolation methods mentioned earlier can lead to varying outcomes, affecting the reproducibility and reliability of sEV-based diagnosis; (2) distinguishing the subpopulation of sEVs poses a challenge; (3) validating these biomarkers and establishing their clinical relevance present difficulties that require rigorous validation studies, large-scale trials, and comparison with existing diagnostic methods. Nevertheless, recent advancements in single-vesicle technologies offer promising potential for the detection of sEVs as biomarkers, rendering them increasingly noninvasive, convenient, and precise [131].

### sEV-based regenerative medicine for pain relief

**Fig. 4:**
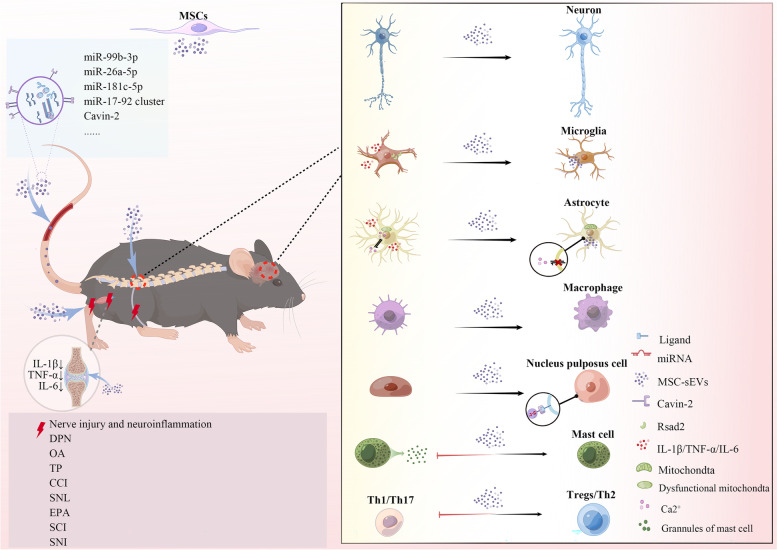
MSC-sEVs for pain relief. MSC-sEVs can be delivered in various ways. The analgesic effects of MSC-sEVs have been verified in various pain models. They promote axonal growth and myelin regeneration, protecting neurons. They alleviate the activation of astrocytes and microglia and promote the transition of M1 to M2 macrophages. Additionally, MSC-sEVs inhibit mast cell degranulation and modulate the Th1/Th17 ratio while promoting Treg cells. MSC-sEVs: mesenchymal stem cell-derived small extracellular vesicle; DPN: diabetic peripheral neuropathy; OA: osteoarthritis; TP: tendinopathy pain; CCI: chronic constriction pain; SNL: spinal nerve ligation; EPA: experimental autoimmune prostatitis; SCI: spinal cord injury; SNI: spared nerve injury

Regenerative medicine has emerged as a promising therapeutic approach for an array of neurological disorders owing to its impressive potential for regeneration and repair, such as spinal cord injury, stroke, Alzheimer’s disease, and traumatic brain injury [132]. Pain management is another area where regenerative medicine has garnered significant attention. MSCs exhibit remarkable diversity in their origins, ranging from an induced pluripotent stem cell (iMSC) to bone marrow (BMSC), umbilical cord (UCMSC), adipose tissue (ADMSC), dental pulp (DMSC), and other sources, all of which have shown remarkable therapeutic efficacy [133]. A growing body of literature suggests that the paracrine effects of sEVs are largely responsible for the therapeutic benefits of MSCs. MSC-derived sEVs (MSC-sEVs) are a promising alternative to traditional cell-based therapies due to their innate advantages over their parent cells. Notably, sEVs offer no immunogenicity and lack neoplastic qualities or differentiation concerns. Additionally, MSC-sEVs are abundant in supply, easy to preserve, and possess the ability to cross biological barriers with ease, such as BBB. Furthermore, sEVs can protect contents against degradation, such as miRNA, and provide multiple delivery modes, making them highly versatile. Indeed, various administration routes, including intravenous, intrathecal, intranasal, and local delivery, have shown efficacy in treatment (Fig. 4) [134–137]. These characteristics demonstrate that MSC-sEVs hold great promise for use in therapeutic applications, particularly in pain relief, and may serve as a substitute for traditional cell-based therapies (Table 2).

According to a recent study, the continuous local delivery of MSC-sEVs has shown promising results in mitigating tendinopathy-associated acute pain in rats over a period of four weeks [137]. The research affirmed that this therapy was capable of hindering mast cell infiltration and reducing the number of positive mast cells surrounding nerve fibers [137]. Furthermore, the administration of MSC-sEVs led to reduced expression of proinflammatory cytokines and lowered activation of mast cells through the hypoxia-inducible factor-1 (HIF-1) signaling pathway, coinciding with an improvement in symptoms [137]. Osteoarthritis (OA) is a typical inflammatory pain. At weeks 2, 4, and 6 in the OA model, the pain threshold was significantly improved after MSC-sEVs treatment [138]. The reduced serum inflammatory mediators including IL-1β, IL-6, and TNF-α, repaired cartilage, and resynthesis of extracellular matrix were associated with the analgesic effects after sEVs application [138]. Furthermore, the anti-inflammatory response and protective effect for chondrocytes were regulated by sEV-encapsulated miRNAs, like miR-23a-3p, miR-9-5p, and miR-396 [139–141]. MiR-23a-3p was found to target the suppression of phosphatase and tensin homologue (PTEN) levels while elevating Protein Kinase B (AKT) expression in cartilage [141]. Moreover, miR-9-5p band to the 3’ untranslated region (3’UTR) of syndecan-1 (SDC1), effectively inhibiting its upregulation. As a result, this inhibition reduced inflammation and mitigated osteoarthritis-like damage [139]. Additionally, miR-3960 could target and inhibit pleckstrin homology-like domain, family A, member 2 (PHLDA2), which was positively associated with SDC1 and activation of the Wnt/β-catenin pathway [140]. It was also reported that MSC-sEVs suppressed glial fibrillary acidic protein (GFAP) and ionized calcium-binding adapter molecule 1 (Iba1) in both the spinal cord and DRG of the SNL pain model. They promoted the pro-inflammatory microglia to restorative type, led to reduced astrocyte activation, and inhibited leucocytes and CD8^+^ T lymphocyte infiltration, all of which contributed to the alleviated neuroinflammation [142].

Apart from inflammatory pain, MSC-sEVs also have the potential ability to alleviate neuropathic pain through diverse intervention modalities. UCMSC-sEVs have dose-dependent analgesic effects on mechanical allodynia and thermal hyperalgesia when intrathecally administered following SNL injury [135]. It was noteworthy that the commencement of action occurred within a mere 15 min. In the group administered with a dosage of 1.2 mg/ml, the rat model exhibited a restoration of normal pain threshold during the initial phase and keep steady pain status at day 8 post-surgery. Additionally, the continuous intrathecal infusion of UCMSC-sEVs for 7 days (hourly dose: 1.2 µg) prevented the development of neuropathic pain, revealing a noteworthy antinociceptive effect [135]. In addition, a single intrathecal injection of MSC-sEVs durably has an analgesic effect on partial sciatic nerve ligation (SNL) and CCI pain model [143–145]. Researchers also adopted a novel approach to apply UCMSC-sEVs. The UCMSC-sEVs were wrapped by a sponge-like alginate scaffold and then the materials were wrapped around ligated nerves in the SNL model [146]. This therapeutic method improved the withdrawal threshold and latency. It was more important that the wrapped sEVs maintained long-lasting antinociceptive effects until day 21 after surgery, better than the single injection. Similarly, the containing-sEVs materials ameliorated the inflammatory microenvironment and promoted the regeneration of axons [146]. This reminded us to pay attention that the extended release of sEVs will improve the therapeutic effects. However, it is noteworthy that the cell supernatant of MSCs was centrifuged at 2000 g and then passed through a 0.22 μm filter to obtain the sEVs [135]. In this study, they found the characterization of sEVs in morphology and marker, but there was no size-distribution analysis. The isolation and extraction of sEVs require multifaceted validation.

PKH26-labeled sEVs were taken up by neurons and microglia in the dorsal horn of the spinal cord [143–145]. The antinociceptive effects were associated with suppressed neuron and glial activation, as well as reduced neuroinflammation. SEVs down-regulated Rsad2 expression and inhibited TLR2/MyD88/NF-κB signaling activation in spinal microglia [143]. In addition, according to miRNA-seq results, miR-26a-5p was enriched in MSC-sEVs and miR-26a-5p alleviated the activation of microglia in spinal cord. It significantly suppressed neuropathic pain and neuroinflammation by targeted modulation of wnt5a in spared nerve injury (SNI) model, contributing to the effect of sEVs [144]. Apart from this, MSC-sEVs have been demonstrated to ameliorate CCI-induced mechanical allodynia and stimulate microglial autophagy by inhibiting the activation of the PI3K/AKT/mTOR signaling pathway [145]. Furthermore, the transfer of miR-99b-3p, miR-190b-5p, and miR-152-3p *via* sEVs significantly suppressed the expression of pro-inflammatory cytokines in activated microglia [145]. The included miRNAs in sEVs, specifically miR-99b-3p, miR-190b-5p, and miR-152-3p, augmented the levels of LC3-II and Beclin-1. Notably, only miR-99b-3p exhibited a substantial reduction in the up-regulation of p62 [145]. The presence of these miRNAs in sEVs is reported to have considerable potential for promoting pain relief by regulating microglia activation. Furthermore, studies have suggested that the axonal growth of neurons is partially attributed to the presence of encapsulated argonaut 2 (Ago2) protein in sEV - a critical effector molecule for miRNAs [28, 147]. The involvement of SNARE is vital for the endocytic uptake of sEVs by neurons [28]. Additionally, MSC-sEVs have regulatory effects on activated astrocytes. Inflammation-induced astrocytes with abnormal calcium signaling and mitochondrial dysfunction were effectively reversed by MSC-sEVs [148].

Diabetic peripheral neuropathy (DPN) often elicits enduring neuropathic pain. The systemic administration of MSC-sEVs every week for a duration of eight weeks was found to have a significant effect on restoring mechanical and thermal thresholds in DPN and promoting the transition of macrophages from an M1 to an M2 phenotype, which in turn curtailed the inflammatory response observed in the sciatic nerves of a murine model [136]. Additionally, these sEVs were also found to enhance nerve conduction velocity in DPN mice [136]. Due to the neuroprotective effect of MSC-sEVs, they have been demonstrated to support the survival and axonal growth of injured neurons in DPN [149]. Remarkably, these sEVs have also been shown to significantly enhance the cell viability of ganglion cells, and promote axon regeneration, and myelination, thereby preventing axonal loss and dysfunction [147, 150–152]. In the peripheral nervous system, the administration of MSC-sEVs was found to facilitate the restoration of motor function and stimulate axon regeneration [153]. They promoted an increase in both the length and number of axons [154]. However, it was reported that exosomes with sizes ranging from 30 to 100 nm exerted a positive impact on neurite outgrowth, while MVs, with sizes between 50 and 1000 nm, exhibited a suppressive effect on the same process [152]. Distinct effects may be observed among various types of sEVs. These effects, while potentially disparate, have yet to be fully elucidated.

Recent research has shed light on the remarkable ability of MSC-sEVs to promote myelination in injured neurons. After being absorbed by Schwann cells (SCs), the MSC-sEVs efficiently prompted SCs proliferation, migration, and release of neurotrophic substances, resulting in improved myelin formation [153, 154]. Furthermore, except for peripheral chronic pain, neurological conditions such as spinal cord injury, stroke, and multiple sclerosis can lead to central pain [2]. To address this challenge, researchers have explored the potential of MSC-sEVs as nanomedicines for central diseases. In the central nervous system, the application of sEVs has also been associated with improved maintenance of myelin. This was evidenced by an increase in the expression of genes involved in myelin production and a rise in the number of oligodendrocytes that actively generated myelin [155]. By delivering their contents, sEVs inhibited neuroinflammation, protected neurons, and promoted myelin sheath growth [156]. These were crucial steps toward restoring neural function and relieving pain in the central nervous system. While preclinical studies have shown promising results, further research is needed to determine the safety and efficacy of MSC-sEVs in central disease use.

MSC-sEVs also appear to be effective in visceral pain. In a recent study, sEVs treatment demonstrated notable alleviation of chronic pelvic pain in the experimental autoimmune prostatitis (EAP) model [146]. Moreover, the sEVs intervention led to a remarkable reversal of Th1 and Th17 cells, alongside an escalation in the count of regulatory T cells (Tregs). Additionally, there was a substantial reduction in COX-2 overexpression which indicated a significant alleviation in the inflammatory state [146]. MSC-sEVs exhibit therapeutic potential for pain associated with IBD and cystitis. These disorders are characterized by sustained inflammatory responses causing tissue damage and neuronal sensitization. Previous research has shown that MSC-sEVs possess anti-inflammatory and immunomodulatory properties, thereby ameliorating IBD and cystitis symptoms. Specifically, MSC-sEVs downregulated the expression of IL-4 and IL-12 in dendritic cells, thereby inhibiting their maturation and differentiation while upregulating the expression of TGF-β [157]. This regulation of T cells was achieved through several mechanisms, including modifying the Th1/Th2 and Th17/Treg ratios [157]. Further studies have demonstrated that the mitochondria and miRNAs in MSC-sEVs promoted the conversion of M1 macrophages to the M2 phenotype, thus contributing to their immunomodulatory effects and potentially mitigating pain induced by IBD or cystitis [158, 159].

### Modified sEVs for pain relief

**Fig. 5:**
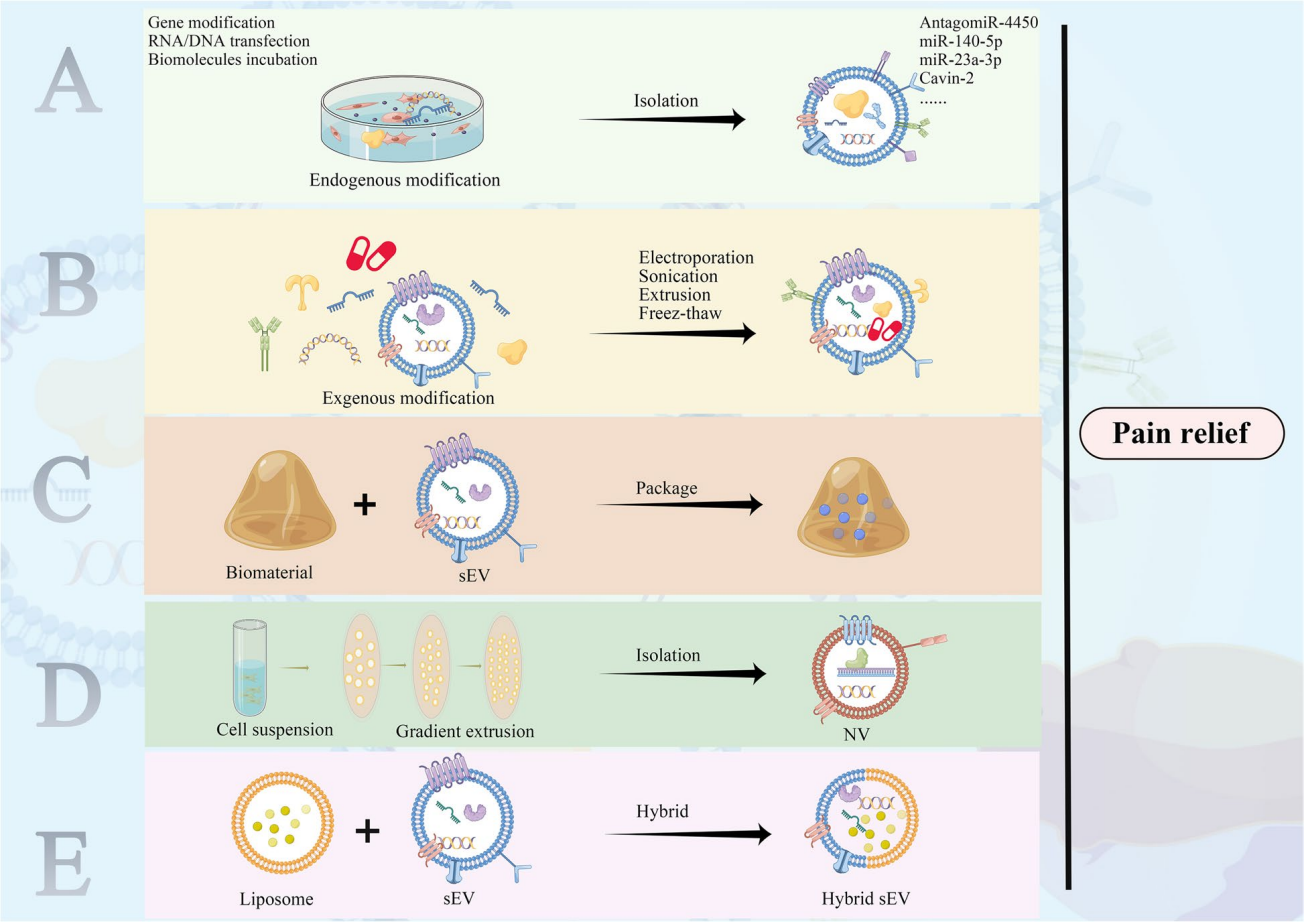
Potential application of modified sEVs for pain relief. The engineered modification of sEVs encompasses both endogenous and exogenous approaches. The integration of sEVs with biomaterials, artificial NVs, and the hybridization of liposomes with sEVs also demonstrate promising analgesic capabilities. sEV: small extracellular vesicle; NV: nanovesicle

 In addition to their therapeutic potential, sEVs can also serve as versatile vehicles for optimized molecule delivery. In recent years, researchers have explored various approaches to modify sEVs to enhance both their efficacy and durability. These techniques include engineered sEVs, the combination of sEVs with biomaterials, the loading of drugs into sEVs, as well as the creation of artificial EVs. Furthermore, these modified sEVs have shown remarkable promise as novel tools for targeted therapy. Currently, the employment of modified sEVs is gaining traction in the field of pain management. This novel approach holds tremendous potential owing to the ability of sEVs to transport bioactive molecules such as proteins, RNAs, lipids, molecules, and drugs to targeted cells effectively. By encapsulating specific therapeutic agents within sEVs and tailoring their properties, researchers seek to optimize the delivery of analgesic factors to dampen pain pathways while minimizing off-target effects. Moreover, sEVs offer several advantages over traditional drug delivery systems, including low immunogenicity, high biocompatibility, and enhanced stability in circulation. The exploitation of modified sEVs in pain management represents a promising avenue for the development of more effective and safer pain therapies (Fig. 5).

sEVs have emerged as promising vehicles for the targeted delivery of drugs and molecules to injured tissues. However, their clinical efficacy is limited by the lack of specificity and targetability. Engineered sEVs to be more specific and targetable can be a feasible solution. On the other hand, by manipulating the composition of these sEVs, researchers can gain insight into the underlying molecular pathways responsible for pain sensation. The engineered approaches involve the modification of producer cells or isolated sEVs. The goals of these methods entail augmenting the generation of sEVs while imparting them with distinct biomolecules. Additionally, the objectives include loading internal lumens or external surfaces of sEVs with pharmacological agents, nucleic acids, and proteins. Furthermore, these proposed modifications aim to facilitate a concentrated deployment of sEVs through heightened targetability. These multifaceted objectives have spurred intensive research efforts toward developing sophisticated techniques for sEV engineering. Such progress is anticipated to enable the design of advanced sEV-based therapeutic and diagnostic modalities with profound implications for personalized medicine. The engineered methods for sEVs can be categorized into two main types: endogenous and exogenous ways. The former method utilizes parent cells as the source of sEVs, which are engineered to alter their contents (Fig. 5A). In contrast, the latter involves the direct modification of isolated sEVs from cells and is commonly employed for loading small RNA and drug molecules (Fig. 5B).

The microenvironmental factors exert a considerable impact on the condition of parent cells, resulting in a modification in the generation of sEVs. This occurrence is particularly notable in vivo, where tissues’ sEVs are susceptible to being impacted by diverse pathological microenvironments. Moreover, the composition of culture media can also affect the quality and quantity of sEVs released by cultured cells. To enhance their bioactivity, different pre-treatment methods have been employed, including exposure to specific biochemical molecules, hypoxia, pH, or temperature variations [160–162]. For example, priming MSCs with TNF-α has been shown to increase the yield of MSC-sEVs and facilitate the polarization of anti-inflammatory M2 macrophages [161]. Investigating the impact of microenvironmental factors in vivo on the characteristics of sEVs could lead to their potential utility as biomarkers for certain disorders, as already discussed above. Another endogenous loading has been extensively employed in prior studies on the use of sEVs for pain disorders. An encouraging approach involves the transfection of nucleic acid therapeutics, including antagomiR-4450 and miR-140-5p, into MSCs *via* recombinant lentivirus-mediated means. The resulting sEVs are then isolated and evaluated for their potential in alleviating aberrant conditions [136, 163, 164]. The miR-4450 was found to upregulate intervertebral discs (IVDs), leading to damage of nucleus pulposus cells (NPCs) through targeted regulation of ZNF121. To combat this, MSC-sEVs loaded with antagomiR-4450 were developed, which improved abnormal gait patterns and attenuated IVD damage by suppressing miR-4450 and increasing ZNF121 expression in an IVD model [163]. Macrophages were transfected with miR-23a-3p after which sEVs enriched in this specific microRNA were extracted. These sEVs, displaying heightened levels of miR-23a-3p, demonstrated remarkable efficacy in mitigating inflammatory pain [97]. Given these promising results, further investigation into loading sEVs with additional miRNAs is warranted to explore the full potential of sEV-based therapies in pain management. On the other hand, surface modification of EVs has recently emerged as a promising approach to augment their targeting and fusion capabilities. In addition, this strategy offers an opportunity to unravel the underlying mechanisms governing sEV functionality. Shedding light on the role of CD200 receptors (CD200r) in sEVs, Michiel et al. have ingeniously engineered macrophages deficient in CD200r, resulting in the isolation of sEVs from these cells for further investigation. Intriguingly, their findings demonstrate that these sEVs were unable to alleviate inflammatory hyperalgesia in mice, thereby underscoring the significance of CD200r in promoting pain resolution by facilitating sEVs’ binding to neurons [21]. The local microenvironment of IVD impaired NPCs’ uptake of MSC-sEVs, highlighting the need for novel approaches to enhance the uptake. Cavin-2 on the membrane was identified as a key regulator of sEV uptake by NPCs. Cavin-2-modified MSC-sEVs were generated *via* gene editing of parental MSCs, which effectively ameliorated NPC cell death in vitro and delayed IVD progression ex vivo [165]. These findings suggest that cavin-2-engineered MSC-sEVs may hold promise as a more effective treatment for low back pain.

Exogenous loading methods involve loading drugs, nucleic acids, or proteins into purified sEVs. Physical approaches such as electroporation, sonication, co-incubation, freeze-thaw, and extrusion have been employed to load a variety of molecules into sEVs. Electroporation has been commonly used to load nucleic acid, protein, and chemotherapeutics into sEVs. For instance, the use of electroporation to load Cas9 RNP protein into hepatic stellate cell-derived sEVs has been reported to target specifically injured liver [166]. Similarly, the miRNA-155 inhibitor was electroporated into B cell-derived sEVs, which showed a significant decrease in LPS-induced miRNA-155 levels in macrophages [167]. The aforementioned molecules, including miR-23a-3p and antagomiR-4450, can also be encapsulated directly into sEVs to improve therapeutic efficiency. Chemotherapeutic agents, such as doxorubicin and paclitaxel (PTX), have been widely loaded into sEVs for tumor treatment [10]. Furthermore, PTX-loaded macrophage-derived sEVs engineered with a ligand by sonication targeted overexpressed sigma receptors on lung cancer cells, achieving a good therapeutic effect for tumors [168]. This targeted approach combining drug loading with engineered sEVs holds great promise for optimizing sEVs treatment, with excellent prospects for much broader and more frequent application. Besides tumor treatment, sEVs and other vesicle analogs have also shown potential in pain management. Liposome-packaged morphine has been demonstrated to prolong the analgesic effect and decrease addictive side reactions, compared with only morphine treatment [169]. The impact of cannabidiol (CBD) as an analgesic agent holds promising potential for pain management. Using monodisperse lipid nanocapsules (LNCs) as biocompatible and biodegradable carriers for CBD encapsulation has proven successful. This innovative approach effectively circumvented dosing concerns associated with cannabinoids and enabled precise regulation of CBD release [170]. As with these investigations, the utilization of sEVs for the encapsulation of analgesics also represents a viable prospect for advancing extended-release drug formulations and reducing the dosage of drugs. SEVs ranging in size from 30 to 200 nm can encase multiple types of hydrophilic drug molecules, while their surface can also be engineered to target neurons or injured tissues specifically. The loaded molecules combine with targetable sEVs to optimize the dose and effect of analgesics, which could potentially offer a safer and more effective approach to pain management. Despite their potential as a promising platform for targeted drug delivery, the loading efficiency of sEVs remains a significant challenge. Loading sEVs through various methods often leads to damage to the vesicles themselves. This factor could potentially account for the limited implementation of engineered extracellular vesicles as a viable solution for pain treatment. Efforts are underway to address this issue by exploring new methods for modifying sEVs that do not adversely affect their integrity. The reported modified sEVs for pain treatment was shown in Table 4.

**Table 4:** Modified sEVs for pain relief

Published year	Model	Resources	Effects	Modified methods	Ref.
2022	Carrageenan-induced pain	M0 macrophages	Resolve inflammatory pain	Artificial vesicles from cells	[21]
2021	IVD	NPs	Anti-catabolic and anti-inflammation	Loaded with FOXF1 by electroporation	[115]
2021	DPN	MSCs	Normalized nerve conduction velocity and compound muscle action potential	Hybrid (fusion of EVs and PpyNps-liposome by freeze-thawing)	[149]
2021	IR-induced pain	Neurons	Attenuated pain hypersensitivity	Hypoxia-precondition induced high level of miR-126-3p	[56]
2021	SNI	Neurons	Reduced neuropathic hypersensitivity	Loaded with miR-21 antagomir	[54]
2020	CFA	M2 macrophages	Increased mechanical allodynia threshold	Transfection with lentivirus-miR-23a-3p	[97]
2020	SNL	MSCs	Upregulated withdrawal threshold and latency	Combination with alginate scaffold	[172]
2017	SNI	Neurons	Attenuated neuropathic hypersensitivity	Loaded with miR-23a antagomir	[53]

*NDEVs *Neuron-derived EVs, *SNI *Spared nerve injury, *CFA *Complete Freund’s Adjuvant, *IR *Ischemia-reperfusion, *SNL *Spinal nerve ligation, *DPN *Diabetic peripheral neuropathy, *PpyNps *Polypyrrole nanoparticles

#### sEVs combine with biomaterials

Although therapeutic sEVs have demonstrated positive effects on various diseases, their administration and dosage greatly diverge. To achieve prolonged effects and direct tissue targeting, researchers have also explored the use of specific biological materials to enhance the therapeutic efficacy of sEVs. Recent studies have focused on the composite application of sEVs with biological biomaterials (Fig. 5C). For instance, photocrosslinkable alginate hydrogels containing fibronectin have been designed to encapsulate, tether, and retain engineered sEVs over a period of seven days while maintaining their structural integrity and functionality [171]. A novel strategy entails the utilization of combinatorial MSC-sEVs that were enmeshed within a porous alginate scaffold to address pain management. This technique was observed to elicit notable analgesic effects in neuropathic pain models, exhibiting a sustained efficacy for up to 21 days. In contrast, single doses of MSC-sEVs were swiftly eliminated and only furnished short-term analgesia, as evidenced by prior investigations [172]. The alginate scaffold delays the release of MSC-sEVs, thereby extending their action time and balancing anti-inflammatory and pro-inflammatory mediators in DRG [172]. Similarly, PDLLA-PEG-PDLLA triblock copolymer gels (PLEL) loaded with circRNA3503-overexpressing MSC-sEVs (PLEL@circRNA3503-OE-sEVs) have shown promise in treating osteoarthritis, particularly in cartilage tissues [173]. The PLEL could slowly release the circRNA3503-loaded sEVs, providing a therapeutic effect [173]. These findings suggest that combining sEVs with biological biomaterials is a promising strategy for improving their therapeutic potential in pain disorders.

#### Biomimetic vesicles

The task of ensuring consistent and optimal encapsulation of therapeutic agents within sEVs remains a significant challenge in the field. Achieving uniform and efficient loading of these agents into sEVs remains an obstacle of considerable importance that warrants further investigation. One of the important reasons is the low production volume of sEVs. Hybrid sEVs, resulting from the fusion of sEVs with liposomes, have emerged as an avenue for addressing this challenge (Fig. 5E). The fusion process, which could be activated by co-extrusion, freeze-thawing, and polyethylene glycol (PEG), resulted in the development of biosynthetic hybrid carriers that combined the advantages of both sEVs and functionalized liposomes [174]. These hybrid sEVs have been demonstrated to be highly effective in augmenting sEVs with exogenous lipophilic or hydrophilic compounds while preserving their innate content and biological properties [175].

On the other hand, nanovesicles (NVs), also known as sEV mimetics, are a type of nanovesicles or nanoparticles that are distinct from sEVs. NVs are obtained from physical methods such as extrusion, sonication, and freeze-thaw, with most of them being derived from cells or liposomes [176, 177]. Recently, microfilters with various pore sizes have been employed to produce NVs mechanically (Fig. 5D). These NVs typically have a diameter ranging from 100 to 250 nm and possess effects that was similar to those of naturally occurring biological sEVs. Interestingly, the yield of NVs was more than 20 times higher than that of sEVs, leading to enhanced bioavailability [178]. NVs, akin to sEVs, exhibit analogous traits concerning their capacity for drug encapsulation and genetic modification [179]. Furthermore, the mechanical stress involved in the extrusion process results in better extensibility of NVs, which may contribute to their improved drug-loading capabilities. It is noteworthy that there are differences in the protein profiles of NVs and sEVs. It was reported that NVs predominantly reflect the proteome of their progenitor cells. In contrast, sEVs exhibit a distinctive protein profile that accentuates their origin from the endosomal compartment [180]. Researchers cultivated 9 different tumor cell lines and collected both sEVs and NVs from these cells. The outcome of sequencing analysis revealed that there was a significant intersection of 71% within the population of 181 membrane proteins presented in both sEVs and NVs. Additionally, the small RNA species within the two compartments revealed over 95% similarity, with the top 1000 small RNAs (smRNAs) displaying a 65% concordance in their expression levels [181]. Moreover, their functional effects are similar. In one study, mechanically extruded NVs derived from MSCs were mixed with an extracellular matrix (ECM) hydrogel to mitigate cardiac injury, achieving similar outcomes as MSC-sEVs [178]. Another study employed microfluidic technology to engineer NVs that expressed neural membrane proteins. These NVs were then administered to human pluripotent stem cell-derived neuron cells, neuron organoid-based spheres, and trigeminal ganglion in vivo, where they exhibited greater affinity and uptake by neurons [176]. It has been demonstrated that artificial vesicles containing mitochondria from M0 macrophages are effective in attenuating inflammatory pain, similar to the effects observed with sEVs separated from cells [21]. It was also reported that MSC-derived NVs displayed neuroprotective properties against injured neuronal cells [182]. NVs present a promising solution for the low yield issue faced by sEVs. It is anticipated that these NVs are likely to become an alternative to sEVs.

It can be speculated that transportation of NVs loading analgesic drugs or specific membrane molecules can effectively promote the repairment of injured neurons while providing pain relief. This innovative method presents a new avenue for therapeutic intervention in the context of pain management and neuronal regeneration. While the actions of NVs are indeed commendable, there remains a need for deeper investigation and comparison between NVs and sEVs, including gene expression, membrane ligands and proteins, and other biomolecules. In the realm of pain management research, the potential of NVs as therapeutic agents remains largely unexplored. While previous studies have highlighted the effectiveness of sEVs in pain relief, a comparative analysis between NVs and EVs is conspicuously absent.

### Prospect and conclusion

The current literature surveyed in this overview centers exclusively on sEVs. According to the report, the utilization of larger electric vehicles (lEVs) resulted in a significant reduction of pain stemming from the TP model, while also regulating the heterogeneity of infiltrated macrophages and various inflammatory cytokines [183]. However, it remains to investigate the other potential properties of lEVs in the pain process, including apoptotic bodies. Further inquiry into the therapeutic benefits of these distinct vesicular populations could provide valuable insights into their clinical applications and expand our understanding of intercellular communication mechanisms. In addition, the extraction of different sEV populations is characterized by a lack of standardization and intrinsic heterogeneity, which in turn results in inconsistent therapeutic dosages across the literature. Comprehensive quantitative analysis of multiple facets of sEVs is crucial following their extraction to facilitate a more rigorous approach.

Currently, observing the real-time visualization of sEVs’ biogenesis, secretion, transport, and mode of action under both physiological and pathological conditions remains a formidable challenge. Imaging of these fundamental processes holds tremendous potential to corroborate and extensively investigate the alterations in targeted tissues in vivo. Thus, it necessitates imaging techniques with high speed, resolution, and sensitivity. Remarkably, zebrafish embryos offer an advantageous platform for non-invasive imaging of sEVs and vascular networks owing to their translucent nature, thereby holding immense promise for detecting even subtle changes [184]. The complexity and heterogeneity of these vesicles present a challenge that requires further exploration in vivo. A novel approach to investigating single vesicles has emerged as a crucial tool in comprehending the pathological under pain condition. The confluence of cutting-edge methodologies such as single vesicle analysis and real-time in vivo observation has opened up new avenues for a more exhaustive exploration of the intricate mechanisms of pain due to sEVs. Despite notable progress, our current understanding of the role played by sEVs in this field is still in its infancy. The study of sEVs has garnered much attention due to their potential as a source of novel biomarkers and therapeutic targets for pain disorders. The mechanisms by which sEVs modulate pain signaling pathways are not yet fully understood, and their use as diagnostic tools requires further validation. Nonetheless, with the continued investigation and technological advancement, we are optimistic that the study of sEVs will yield valuable insights into the nature of pain and contribute to the development of effective treatments. However, the potential of modified sEVs as an analgesic therapy remains largely unexplored, despite promising initial findings. Further investigation into cargo and targeted modifications are necessary to unlock the full therapeutic potential of sEVs in pain management.

The preliminary establishment of the role of sEVs in the pathogenesis, diagnosis, and treatment of pain has been observed. However, several significant gaps in knowledge still exist, indicating a requirement for a more profound comprehension of the underlying mechanisms. Thus, it is imperative to carry out extensive research on the effects of sEVs on pain to unleash their complete potential. This initiative will pave the way for innovative and less invasive therapies for pain management that may be more effective than conventional treatments.

## Data Availability

Not applicable.

## Abbreviations

sEV: Small extracellular vesicle
Exos: Exosomes
MVs: Microvesicles
Abs: Apoptotic bodies
MVB: Multivesicular body
ILV: Intraluminal vesicle
MSC: Mesenchymal stem cell
BBB: Blood-brain barrier
BSB: Blood-spinal cord barrier
LTP: Long-term potentiation
Syt4: Synaptotagmin 4
NDEVs: Neuron-derived small extracellular vesicles
GluR2: Glutamate receptor-2
DRG: Dorsal root ganglion
miRNA: microRNA
TRPV-1: Transient Receptor Potential Vanilloid 1
CNS: Central nervous system
IL-1: Interleukin-1
MDEV: Microglia-derived small extracellular vesicle
SNL: Spinal nerve ligation
CSF: Cerebrospinal fluid
PWT: Paw withdrawal threshold
PWL: Paw withdrawal latency
mIPSC: Miniature inhibitory postsynaptic current
sEPSC: Spontaneous excitatory postsynaptic current
eCBs: Endocannabinoids
AEA: N-arachidonoylethanolamine
CB1: Type-1 cannabinoid receptors
GLT1: Glutamate transporter-1
ADEVs: Astrocyte-derived small extracellular vesicle
ApoD: Apolipoprotein D
NCAM: Neural cell adhesion molecule
IL-10: Interleukin-10
rSC: Repair Schwann cell
dSC: Differentiated Schwann cell
SDEV: Schwann cell-derived extracellular vesicles
SGC: Satellite glial cell
mtDNA: mitochondrial DNA
mtRNA: mitochondrial RNA
CFA: Complete Freund’s Adjuvant
LPS: Lipopolysaccharide
SNI: Spared nerve injury
C5a: Complement component 5a
ICAM-1: Intercellular Adhesion Molecule-1
CRPS: Complex regional pain syndrome
IL-6: Interleukin 6
IVD: Intervertebral disc
NP: Nucleus pulposus
NPEVs: Nucleus pulposus-derived small EVs
FOXF1: Forkhead box F1
IBD: Inflammatory bowel disease
PAMPs: Pathogen-Associated Molecular Patterns
MSC-sEVs: Mesenchymal stem cell-derived small extracellular vesicle
ATXN1: Ataxin 1
Ccl3: Chemokine ligand 3
OA: Osteoarthritis
HIF-1: Hypoxia-inducible factor-1
SNARE: Soluble N-ethylmaleimide sensitive factor attachment protein receptor
Ago2: Argonaut 2
SCs: Schwann cells
EPA: Experimental autoimmune prostatitis
DCs: Dendritic cells
Tregs: Regulatory T cells
NVs: Nanovesicles
GPCR: G protein-coupled receptor
CLR: Calcitonin receptor-like receptor
NK1R: Neurokinin 1 receptor
PTX: Paclitaxel
CBD: Cannabidiol
OPRM1: 1 opioid receptor
PEG: Polyethylene glycol
ECM: Extracellular matrix
smRNA: Small RNA
GFAP: Glial fibrillary acidic protein
Iba1: Ionized calcium-binding adapter molecule 1
PRR7: Proline-rich 7
PSD-95: Postsynaptic density-95
vGLUT1: Vesicular glutamate transporter 1
ATX: Autotaxin
LPA: Enzyme synthetizing lysophosphatidic acid
LPAR: LPA receptor
MAPK: Mitogen-activated protein kinase
NF-B: Nuclear factor B
